# Supplementary material for: Living through the heat: How urban children and young people experience and envision healthier cities
Source: PLOS Glob Public Health. 2025 Oct 29;5(10):e0004879. doi: 10.1371/journal.pgph.0004879 (PMC12571289; doi:10.1371/journal.pgph.0004879)
Supplement: S1 File — Describes the criteria and process used to identify and select the 178 focal cities used for the event identification in the study. (DOCX) [file pgph.0004879.s001.docx]

# **Supplementary Information (S) 1 File: Protocol for Selecting the CCC Action Lab Focal Cities**

**1. Objective**

The objective of this protocol is to outline the criteria and methodology used for selecting the 179 focal cities for the Children, Cities, and Climate (CCC) Real-Time research. The selection aimed to represent diverse global perspectives, focusing on the youngest and most rapidly urbanizing cities to align with the goals of the CCC Action Lab and its partner organizations.

**2. Data Sources**

The selection process utilized various credible data sources to identify the target cities:

- **World Bank’s Demographic Trends and Urbanization Report^^[[1]](#footnote-0)^^:** For cities in East Asia and the Pacific, Eastern Europe and Central Asia, Latin America and the Caribbean, the Middle East and North Africa, and South Asia.
- **UN Population Division Data^^[[2]](#footnote-1)^^:** Collated by the African Cities Research Consortium for cities in Africa.
- **CCC Action Lab Partner Contributions:** Including data from C40, Urban 95 cities (developed by the Bernard van Leer Foundation), and the Botnar Foundation.

**3. Selection Criteria**

Each city selected had to meet specific criteria:

- **Population Size:** Cities with a population over 50,000.
- **Urbanization Rate:** Focus on the most rapidly urbanizing cities.
- **Youth Population:** Cities with a significant proportion of young residents.

**4. Selection Methodology**

**4.1. Geographic Regions and Data Sources**

1. **East Asia and the Pacific:**

- Data Source: World Bank’s Demographic Trends and Urbanization report.
- Selection Focus: Youngest and most rapidly urbanizing cities.
- Cities include Beijing (China), Ho Chi Minh (Vietnam), Manila (Philippines), Tokyo (Japan)

1. **Eastern Europe and Central Asia:**

- Data Source: World Bank’s Demographic Trends and Urbanization report.
- Selection Focus: Youngest and most rapidly urbanizing cities.
- Cities Include: Minsk (Belarus), Kiev (Ukraine), Sofia (Bulgaria), Zagreb (Croatia), Tallinn (Estonia)

1. **Latin America and the Caribbean:**

- Data Source: World Bank’s Demographic Trends and Urbanization report.
- Selection Focus: Youngest and most rapidly urbanizing cities.
- Cities Include: Manaos (Brazil), Panama (Panama), RM Lima (Peru), Gran Santiago (Chile), Guadalajara (Mexico)

1. **Middle East and North Africa:**

- Data Source: World Bank’s Demographic Trends and Urbanization report.
- Selection Focus: Youngest and most rapidly urbanizing cities.
- Cities Include: Amman (Jordan), Greater Tehran (Iran), Algiers (Algeria), Cairo (Egypt).

1. **South Asia:**

- Data Source: World Bank’s Demographic Trends and Urbanization report.
- Selection Focus: Youngest and most rapidly urbanizing cities.
- Cities Include: Kathmandu District (Nepal), Thimphu Municipality (Bhutan), Colombo District (Sri Lanka), Dhaka Metropolitan Area (Bangladesh), Mumbai Municipal corporation (India)

1. **Africa:**

- Data Source: UN Population Division data, collated by the African Cities Research Consortium.
- Selection Focus: Twenty fastest urbanizing and youngest cities.
- Cities Include: Abidjan (Côte d'Ivoire), Abuja (Nigeria), Addis Ababa (Ethiopia), Al-Iskandariyah/Alexandria (Egypt), Al-Khartum/Khartoum (Sudan)

**4.2. Partner Contributions**

- **Phase 1 Cities:**
- Example Cities: Bhubaneswar (India), Dar es Salaam (Tanzania), Dhaka (Bangladesh).
- **U95 Cities:**
- Example Cities: Addis Ababa (Ethiopia), Agartala (India), Alcinópolis (Brazil).
- **C40 Cities:**
- Example Cities: Abidjan (Côte d’Ivoire), Accra (Ghana), Addis Ababa (Ethiopia).
- **Botnar Focal Cities:**
- Example Cities: Ashaiman (Ghana), Bandung (Indonesia), Barranquilla (Colombia).

Detailed list of cities categorized by partner organizations, youngest and urbanized cities per region and phases is shown in the table below.

| **Urban95 Cities** | **C40** | **Fondation Botnar's Focal Cities** |
| --- | --- | --- |
| Addis Ababa (Ethiopia) | Abidjan, Côte d’Ivoire | Ashaiman, Ghana |
| Agartala (India) | Accra, Ghana | Bandung (Indonesia) |
| Alcinópolis (Brazil)​ | Addis Ababa, Ethiopia | Barranquilla (Colombia) |
| Alfenas (Brazil)​ | Amsterdam, The Netherlands | Cali (Colombia) |
| Amman (Jordan) | Athens, Greece | Cluj (Romania) |
| Amsterdam (The Netherlands) ​ | Auckland, New Zealand | Envigado (Colombia) |
| Aracaju (Brazil)​ | Bangkok, Thailand | Manta (Ecuador) |
| Arequipa (Perú) | Barcelona, Spain | Tamale, Ghana |
| Ashdod (Israel) | Beijing, China | Tanga (Tanzania) |
| Azraq (Jordan)​ | Berlin, Germany | Thiès, Senegal |
| Barranquilla (Colombia) | Cape Town, South Africa |  |
| Benevides (Brazil) | Chengdu, China | **20 African Cities** |
| Bengaluru (India) | Copenhagen, Denmark | Abidjan (Côte d'Ivoire)​ |
| Bet Shemesh (Israel) | Dakar, Senegal | Abuja (Nigeria)​ |
| Bhubaneswar (India)​ | Dalian, China | Addis Ababa (Ethiopia)​ |
| Boa Vista (Brazil)​ | Dar es Salaam, Tanzania | Al-Iskandariyah/Alexandria (Egypt)​ |
| Bogota (Colombia)​ | Durban (eThekwini), South Africa | Al-Khartum/Khartoum (Sudan)​ |
| Brasileia (Brazil)​ | Ekurhuleni, South Africa | Al-Qahirah/Cairo (Egypt)​ |
| Cali (Colombia) | Freetown, Sierra Leone | Antananarivo (Madagascar)​ |
| Callao (Perú) | Fuzhou, China | Cape Town (South Africa)​ |
| Campinas (Brazil)​ | Guangzhou, China | Dar es Salaam (Tanzania) ​ |
| Canoas (Brazil)​ | Hangzhou, China | Douala (Cameroon)​ |
| Cartagena (Colombia) | Hanoi, Vietnam | Johannesburg (South Africa)​ |
| Caruaru (Brazil)​ | Heidelberg, Germany | Kampala (Uganda)​ |
| Cascavel (Brazil) | Ho Chi Minh City, Vietnam | Kinshasa (DR. Congo)​ |
| Coimbatore (India) | Hong Kong, China | Kumasi (Ghana)​ |
| Crato (Brazil)​ | Istanbul, Turkey | Lagos (Nigeria)​ |
| Cusco (Perú) | Jakarta, Indonesia | Luanda (Angola) ​ |
| Dharamshala (India) | Johannesburg, South Africa | Nairobi (Kenya)​ |
| Erode (India) | Kuala Lumpur, Malaysia | Ouagadougou (Burkina Faso)​ |
| Fortaleza (Brazil)​ | Lagos, Nigeria | Port Harcourt (Nigeria)​ |
| Hubballi - Dharwad (India) | Lisbon, Portugal | Yaoundé (Cameroon)​ |
| Hyderabad (India) | London, United Kingdom |  |
| Illheus (Brazil)​ | Madrid, Spain | **Youngest and most urban** |
| Ilo (Perú) | Melbourne, Australia | ***East Asia and Pacific*** |
| Indore (India) | Milan, Italy | Beijing (China) |
| Irbid (Jordan) | Nairobi, Kenya | Ho Chi Minh (Vietnam) |
| Istanbul (Turkey)​ | Nanjing, China | Manila (Philippines) |
| Izmir (Turkey) | Oslo, Norway | Tokyo (Japan) |
| Jabalpur (India) | Paris, France | ***Eastern Europe and Central Asia*** |
| Jaen (Perú) | Qingdao, China | Minsk (Belarus) |
| Jundiai (Brazil)​ | Quezon City, Philippines | Kiev (Ukraine) |
| Kakinada (India) | Rome, Italy | Sofia (Bulgaria) |
| Kochi (India) | Rotterdam, The Netherlands | Zagreb (Croatia) |
| Kohima (India) | Seoul, Republic of Korea | Tallinn (Estonia) |
| Kota (India) | Shanghai, China | ***Latin America and the Caribbean*** |
| La Pintana (Chile) | Shenzhen, China | Manaos (Brazil) |
| Lima (Peru)​ | Singapore, Singapore | Panama (Panama) |
| Mixco (Guatemala) | Stockholm, Sweden | RM Lima (Peru) |
| Mogi das Cruzes (Brazil) | Sydney, Australia | Gran Santiago (Chile) |
| Monterrey (Mexico) | Tel Aviv - Yafo, Israel | Guadalajara (Mexico) |
| Nagpur (India) | Tokyo, Japan | ***Middle East and North Africa*** |
| Niteroi (Brazil)​ | Tshwane, South Africa | Amman (Jordan) |
| Paragominas (Brazil) | Warsaw, Poland | Greater Tehran (Iran) |
| Pelotas (Brazil)​ | Wuhan, China | Algiers (Algeria) |
| Piura (Peru)​ | Yokohama, Japan | Cairo (Egypt) |
| Pune (India) | Zhenjiang, China | ***South Asia*** |
| Rajkot (India) |  | Kathmandu District (Nepal) |
| Ranchi (India) | **16 Phase 1 cities** | Thimphu Municipality (Bhutan) |
| Recife (Brazil) | Bhubaneswar (India)​ | Colombo District (Sri Lanka) |
| Rohtak (India) | Dar es Salaam (Tanzania)​ | Dhaka (Bangladesh) |
| Rotterdam (The Netherlands) | Dhaka (Bangladesh) | Mumbai (India) |
| Rourkela (India) | Free Town (Sierra Leone)​ |  |
| Salem (India) | Glasgow (English)​ |  |
| São José dos Campos  (Brazil) | Harare (English)​ |  |
| Sao Paolo (Brazil)​ | Jaipur (India) |  |
| Sobral (Brazil) | Lahore (Pakistan)​ |  |
| Surat (India) | London (United Kingdom)​ |  |
| Tel Aviv (Israel)​ | Los Angeles (USA)​ |  |
| Teresina (Brazil) | Mexico City (Mexico)​ |  |
| Thiruvananthapuram (India) | Milan (Italy)​ |  |
| Tira (Israel) | Nairobi (Kenya)​ |  |
| Tirana (Albania)​ | Quezon City (Philippines)​ |  |
| Tiruppur (India) | Quito (Ecuador)​ |  |
| Ubirata (Brazil)​ | Tamale (Ghana)​ |  |
| Udaipur (India) |  |  |
| Ujjain (India) |  |  |
| Uruçuca (Brazil) |  |  |
| Vadodara (India) |  |  |
| Warangal (India) |  |  |

**6. Review and Approval**

The selection protocol and the final list of cities were reviewed and approved by the CCC Action Lab’s Members, ensuring deduplication and alignment with the research's objectives and partner interests.

| **City (Country)** | | |
| --- | --- | --- |
| Abidjan (Côte d'Ivoire)​ | Fortaleza (Brazil)​ | Nairobi (Kenya)​ |
| Abuja (Nigeria)​ | Free Town (Sierra Leone)​ | Nanjing, China |
| Accra, Ghana | Fuzhou, China | Niteroi (Brazil)​ |
| Addis Ababa (Ethiopia) | Glasgow (English)​ | Oslo, Norway |
| Agartala (India) | Gran Santiago (Chile) | Ouagadougou (Burkina Faso)​ |
| Alcinópolis (Brazil)​ | Greater Tehran (Iran) | Panama (Panama) |
| Alfenas (Brazil)​ | Guadalajara (Mexico) | Paragominas (Brazil) |
| Algiers (Algeria) | Guangzhou, China | Paris, France |
| Al-Iskandariyah/Alexandria (Egypt)​ | Hangzhou, China | Pelotas (Brazil)​ |
| Al-Khartum/Khartoum (Sudan)​ | Hanoi, Vietnam | Piura (Peru)​ |
| Amman (Jordan) | Harare (Zimbabwe)​ | Port Harcourt (Nigeria)​ |
| Amsterdam (The Netherlands) ​ | Heidelberg, Germany | Pune (India) |
| Antananarivo (Madagascar)​ | Ho Chi Minh (Vietnam) | Qingdao, China |
| Aracaju (Brazil)​ | Hong Kong, China | Quezon City (Philippines)​ |
| Arequipa (Perú) | Hubballi - Dharwad (India) | Quito (Ecuador)​ |
| Ashaiman, Ghana | Hyderabad (India) | Rajkot (India) |
| Ashdod (Israel) | Illheus (Brazil)​ | Ranchi (India) |
| Athens, Greece | Ilo (Perú) | Recife (Brazil) |
| Auckland, New Zealand | Indore (India) | Rohtak (India) |
| Azraq (Jordan)​ | Irbid (Jordan) | Rome, Italy |
| Bandung (Indonesia) | Istanbul (Turkey)​ | Rotterdam (The Netherlands) |
| Bangkok, Thailand | Izmir (Turkey) | Rourkela (India) |
| Barcelona, Spain | Jabalpur (India) | Salem (India) |
| Barranquilla (Colombia) | Jaen (Perú) | São José dos Campos  (Brazil) |
| Beijing, China | Jaipur (India) | Sao Paolo (Brazil)​ |
| Benevides (Brazil) | Jakarta, Indonesia | Seoul, Republic of Korea |
| Bengaluru (India) | Johannesburg (South Africa)​ | Shanghai, China |
| Berlin, Germany | Jundiai (Brazil)​ | Shenzhen, China |
| Bet Shemesh (Israel) | Kakinada (India) | Singapore, Singapore |
| Bhubaneswar (India)​ | Kampala (Uganda)​ | Sobral (Brazil) |
| Boa Vista (Brazil)​ | Kathmandu District (Nepal) | Sofia (Bulgaria) |
| Bogota (Colombia)​ | Kiev (Ukraine) | Stockholm, Sweden |
| Brasileia (Brazil)​ | Kinshasa (DR. Congo)​ | Surat (India) |
| Cairo (Egypt) | Kochi (India) | Sydney, Australia |
| Cali (Colombia) | Kohima (India) | Tallinn (Estonia) |
| Callao (Perú) | Kota (India) | Tamale (Ghana)​ |
| Campinas (Brazil)​ | Kuala Lumpur, Malaysia | Tanga (Tanzania) |
| Canoas (Brazil)​ | Kumasi (Ghana)​ | Tel Aviv (Israel)​ |
| Cape Town, South Africa | La Pintana (Chile) | Teresina (Brazil) |
| Cartagena (Colombia) | Lagos (Nigeria)​ | Thiès, Senegal |
| Caruaru (Brazil)​ | Lahore (Pakistan)​ | Thimphu Municipality (Bhutan) |
| Cascavel (Brazil) | Lima (Peru)​ | Thiruvananthapuram (India) |
| Chengdu, China | Lisbon, Portugal | Tira (Israel) |
| Cluj (Romania) | London (United Kingdom)​ | Tirana (Albania)​ |
| Coimbatore (India) | Los Angeles (USA)​ | Tiruppur (India) |
| Colombo District (Sri Lanka) | Luanda (Angola) ​ | Tokyo (Japan) |
| Copenhagen, Denmark | Madrid, Spain | Tshwane, South Africa |
| Crato (Brazil)​ | Manaos (Brazil) | Ubirata (Brazil)​ |
| Cusco (Perú) | Manila (Philippines) | Udaipur (India) |
| Dakar, Senegal | Manta (Ecuador) | Ujjain (India) |
| Dalian, China | Melbourne, Australia | Uruçuca (Brazil) |
| Dar es Salaam (Tanzania)​ | Mexico City (Mexico)​ | Vadodara (India) |
| Dhaka (Bangladesh) | Milan (Italy)​ | Warangal (India) |
| Dharamshala (India) | Minsk (Belarus) | Warsaw, Poland |
| Douala (Cameroon)​ | Mixco (Guatemala) | Wuhan, China |
| Durban (eThekwini), South Africa | Mogi das Cruzes (Brazil) | Yaoundé (Cameroon)​ |
| Ekurhuleni, South Africa | Monterrey (Mexico) | Yokohama, Japan |
| Envigado (Colombia) | Mumbai (India) | Zagreb (Croatia) |
| Erode (India) | Nagpur (India) | Zhenjiang, China |

1. https://documents.worldbank.org/en/publication/documents-reports/documentdetail/260581617988607640/demographic-trends-and-urbanization [↑](#footnote-ref-0)
2. https://population.un.org/wpp/ [↑](#footnote-ref-1)
