# Supplementary material for: Living through the heat: How urban children and young people experience and envision healthier cities
Source: PLOS Glob Public Health. 2025 Oct 29;5(10):e0004879. doi: 10.1371/journal.pgph.0004879 (PMC12571289; doi:10.1371/journal.pgph.0004879)
Supplement: S2 File — Includes screenshots and copies of the online recruitment advertisements used in each of the six study cities. (DOCX) [file pgph.0004879.s002.docx]

**Supplementary Information (S) 2 File: Overview of Survey Advertisement Strategies and Visual Campaigns Across Six Cities**

Bwire, C (2025). Overview of Survey Advertisement Strategies and Visual Campaigns Across Six Cities. [Data Collection]. London School of Hygiene & Tropical Medicine, London, United Kingdom. https://doi.org/10.17037/DATA.00004689.
