# Supplementary material for: Living through the heat: How urban children and young people experience and envision healthier cities
Source: PLOS Glob Public Health. 2025 Oct 29;5(10):e0004879. doi: 10.1371/journal.pgph.0004879 (PMC12571289; doi:10.1371/journal.pgph.0004879)
Supplement: S4 File — Contains the anonymized, cleaned dataset used for quantitative analysis in this study. (DOCX) [file pgph.0004879.s004.docx]

**Supplementary Information (S) 4 File: Quantitative Dataset.**

[**Bwire, C**](https://datacompass.lshtm.ac.uk/view/creators/8994b722db9c33efd933c795fe59f51f.html) (2025). *Impact of Heatwave on Children and Young People: Dataset from Six Cities.* [Data Collection]. London School of Hygiene & Tropical Medicine, London, United Kingdom. <https://doi.org/10.17037/DATA.00004617>.
