# Supplementary material for: Living through the heat: How urban children and young people experience and envision healthier cities
Source: PLOS Glob Public Health. 2025 Oct 29;5(10):e0004879. doi: 10.1371/journal.pgph.0004879 (PMC12571289; doi:10.1371/journal.pgph.0004879)
Supplement: S5 File — Outlines the planned analytical approach for both quantitative and qualitative data. (DOCX) [file pgph.0004879.s005.docx]

**Supplementary Information (S)5 File: Data analysis plan**

[**Bwire, C**](https://datacompass.lshtm.ac.uk/view/creators/8994b722db9c33efd933c795fe59f51f.html), Hughes, R, [**Milner, J**](https://datacompass.lshtm.ac.uk/view/creators/a05a5acd6cbe4fb0746f8c81c533e4bc.html), Yeung, S, Bonell, A and [**Bonnet, G**](https://datacompass.lshtm.ac.uk/view/creators/e68e8d8c9e61947d62a58c98eeac979a.html) (2025). *Data Analysis Plan for Event (Heatwave and High Air Pollution) and Non-Event (Average Temperature and Moderate/Good Air Quality) Survey.* [Data Collection]. London School of Hygiene & Tropical Medicine, London, United Kingdom. <https://doi.org/10.17037/DATA.00004653>.
