# Supplementary material for: Living through the heat: How urban children and young people experience and envision healthier cities
Source: PLOS Glob Public Health. 2025 Oct 29;5(10):e0004879. doi: 10.1371/journal.pgph.0004879 (PMC12571289; doi:10.1371/journal.pgph.0004879)
Supplement: S6 File — Provides an example of the outreach email used to inform city officials of the data collection in their city. (DOCX) [file pgph.0004879.s006.docx]

# **Supplementary Information (S) 6 File: Sample Mayor’s Email**

**Dear (Mayor’s Name),**

I hope this message finds you well. I am writing to inform you that (city name) has been identified for inclusion in a research research conducted by the Children, Cities and Climate (CCC) Action Lab. The CCC Action Lab studies the health and well-being of Children, Young People, and Parents in cities affected by extreme climate change related events.

[**About the Children, Cities, and Climate Action Lab**](https://www.lshtm.ac.uk/research/centres-projects-groups/children-cities-and-climate) The Action Lab, led by [London School of Hygiene and Tropical Medicine](https://www.lshtm.ac.uk/), in partnership with [C40 Cities](https://www.c40.org/), [YLabs](https://www.ylabsglobal.org/), and [UrbanBetter](https://urbanbetter.science/). This consortium combines expertise in public health, advocacy, youth engagement, and climate resilience to tackle some of the most pressing challenges facing urban populations today. Our goal is to integrate scientific research with actionable policy solutions, enhancing the livability and sustainability of cities around the world.

**Purpose of this research:** To understand the impact of extreme climate change related events, i.e., heatwaves on the health, well-being, and disruptions to the daily life of children, young people, and parents in cities to generate evidence that can guide effective urban planning and policy.

**Why (City Name)?** (City Name) has been selected as a key participant due to its vulnerability to climate change related events i.e., heatwaves and C40’s current collaborations with your city. The city’s involvement would not only provide invaluable insights into the local impacts of global climate change but also highlight (City Name)'s role as a leader in urban climate resilience.

**Survey Implementation and Data Sharing** In the upcoming weeks, we will initiate global online surveys conducted through Facebook and Instagram, targeting residents in (City Name) and other affected cities worldwide. By December 2024, we intend to release a detailed report outlining our collective findings, with specific data relevant to (City Name). We are keen to share city specific results with your office and discuss ways they can inform local policy decisions after data collection, by September 2024 through various formats that suit your preferences, including direct reports, webinars, or interactive virtual consultations.

Thank you very much for your time. We believe that together, we can make significant strides towards a more sustainable and resilient future for (City Name). I am available at your convenience to answer any questions you might have.

**Yours sincerely,**

LSHTM representative

on behalf of Dr. Rob Hughes,
Assistant Professor,
London School of Hygiene & Tropical Medicine
