# Supplementary material for: Living through the heat: How urban children and young people experience and envision healthier cities
Source: PLOS Glob Public Health. 2025 Oct 29;5(10):e0004879. doi: 10.1371/journal.pgph.0004879 (PMC12571289; doi:10.1371/journal.pgph.0004879)
Supplement: S1 Fig — Illustrates the process for identifying of eligible heatwave and non-heatwave periods and participant recruitment in the six cities. (DOCX) [file pgph.0004879.s007.docx]

**Supplementary Information (S)1 Fig.:** Heatwave Event and Non-Heatwave Event Sampling Flowchart Automated Identification, Data Collection, and Monitoring Protocol.

Retrieved from: [**Bwire, C**](https://datacompass.lshtm.ac.uk/view/creators/8994b722db9c33efd933c795fe59f51f.html), [**Juel, R**](https://datacompass.lshtm.ac.uk/view/creators/d60f74bed54bf620b67406ada8ea6c2f.html), [**Hughes, R**](https://datacompass.lshtm.ac.uk/view/creators/9404f39cae6a401b9e26d483e7b1c580.html), [**Yeung, S**](https://datacompass.lshtm.ac.uk/view/creators/4486dea5b88662a0fa3e13aff5359590.html), [**Bonell, A**](https://datacompass.lshtm.ac.uk/view/creators/f10fa1ec89e261ec7b0ff58b99092576.html) and [**Milner, J**](https://datacompass.lshtm.ac.uk/view/creators/a05a5acd6cbe4fb0746f8c81c533e4bc.html) (2025). *Heatwave Event and Non-Heatwave Event Sampling Flowchart Automated Identification, Data Collection, and Monitoring Protocol.* [Data Collection]. London School of Hygiene & Tropical Medicine, London, United Kingdom. <https://doi.org/10.17037/DATA.00004616>.
