# Supplementary material for: Living through the heat: How urban children and young people experience and envision healthier cities
Source: PLOS Glob Public Health. 2025 Oct 29;5(10):e0004879. doi: 10.1371/journal.pgph.0004879 (PMC12571289; doi:10.1371/journal.pgph.0004879)
Supplement: S2 Table — Summarizes key metrics on ad impressions, click-throughs, and completed surveys by city. (DOCX) [file pgph.0004879.s009.docx]

**Supplementary Information (S) 2 Table: Ad Performance and Survey Engagement Metrics During Heatwave and Non-Heatwave Periods Across the Six Study Cities**

Table 1: Ad Performance and Survey Engagement Metrics During Heatwave and Non-Heatwave Periods Across the Six Study Cities

| City | Event | CTR (%) | CPC (£) | Clicks | Impressions | Start Date | Days Run | Temperature (OC) | Survey Views | Survey Starts | Submitted Surveys | Completion Rates (%) | Eligible Surveys |
| --- | --- | --- | --- | --- | --- | --- | --- | --- | --- | --- | --- | --- | --- |
| Ouagadougou | Heatwave | 5.23 | 0.04 | 4530 | 86600 | 21/10/2024 | 5 | 35.1 - 24.2 | 7,237 | 2,321 | 446 | 19.2 | 187 |
|  | Non- Heatwave | 4.61 | 0.03 | 2770 | 60000 | 7/11/2024 | 7 | 25.4 | 4,045 | 1,309 | 178 | 13.6 | 57 |
| Kumasi | Heatwave | 2.53 | 0.04 | 3630 | 144000 | 19/10/2024 | 7 | 36.2 - 26.4 | 6,329 | 1,763 | 462 | 26.2 | 270 |
|  | Non- Heatwave | 3.09 | 0.04 | 482 | 13400 | 07/11/2024 | 9 | 25.0 | 367 | 106 | 51 | 48.1 | 23 |
| Accra | Heatwave | 2.01 | 0.03 | 5420 | 270000 | 24/10/2024 | 7 | 36.6 - 28.1 | 7981 | 2,652 | 774 | 20.75 | 486 |
|  | Non- Heatwave | 2.11 | 0.06 | 1710 | 80800 | 4/11/2024 | 9 | 26.6 | 1,538 | 461 | 218 | 47.3 | 75 |
| Manilla | Heatwave | 4.20 | 0.02 | 104,300 | 382000 | 11/10/2024 | 7 | 40.1 - 29.1 | 28,911 | 3,448 | 820 | 20.75 | 362 |
|  | Non- Heatwave | 7.32 | 0.02 | 6300 | 86000 | 13/11/2024 | 9 | 26.6 | 6,161 | 2,660 | 316 | 11.9 | 85 |
| Port Harcourt | Heatwave | 3.10 | 0.04 | 9490 | 134000 | 23/10/2024 | 4 | 38.2 - 29.2 | 5,989 | 1,633 | 502 | 30.7 | 312 |
|  | Non- Heatwave | 2.8 | 0.03 | 1204 | 7300 | 28/10/2024 | 9 | 24.9 | 1160 | 96 | 43 | 44.8 | 13 |
| Dar es Salaam | Heatwave | 17.5 | 0.02 | 6350 | 36200 | 30/10/2024 | 4 | 36.4 - 30.3 | 6,659 | 3,330 | 781 | 23.5 | 171 |
|  | Non- Heatwave | 14.6 | 0.02 | 5620 | 38400 | 06/11/2024 | 4 | 26.7 | 9,536 | 3,347 | 828 | 24.7 | 223 |

This table compares digital advertisements from Google and Meta, and survey performance across six cities during heatwave and non-heat wave periods. Key indicators include click-through rates (CTR), cost-per-click (CPC), survey views, starts, submissions, and completion rates. The data show variation in public engagement across cities and event types.
