# Supplementary material for: Living through the heat: How urban children and young people experience and envision healthier cities
Source: PLOS Glob Public Health. 2025 Oct 29;5(10):e0004879. doi: 10.1371/journal.pgph.0004879 (PMC12571289; doi:10.1371/journal.pgph.0004879)
Supplement: S3 Table — Provides a breakdown of age, gender, and parental status for survey respondents. (DOCX) [file pgph.0004879.s010.docx]

**Supplementary Information (S) 3 Table: Cross-tabulations and Statistical Analyses of Demographic Variables During Heatwave and Non-Heatwave Periods Across Six Cities**

*Table 1: Cross-tabulation of Parental Status during Heatwave and Non-Heatwave periods with Chi-Square Test Results Across Six Cities*

| **City** | **Cross-tabulations** | | | | | **Chi-Square Tests** | | | |
| --- | --- | --- | --- | --- | --- | --- | --- | --- | --- |
|  |  |  | Event | | Total |  |  |  |  |
|  |  |  | Heatwave | Non-Heatwave |  |  | Value | df | Asymptotic Significance (2-sided) |
| Accra | Parent | No | 248 | 34 | 282 | Pearson Chi-Square | .843c | 1 | 0.359 |
|  |  | Yes | 238 | 41 | 279 | Likelihood Ratio | 0.844 | 1 | 0.358 |
|  | Total |  | 486 | 75 | 561 | N of Valid Cases | 561 |  |  |
| Dar es Salaam | Parent | No | 79 | 118 | 197 | Pearson Chi-Square | 1.207d | 1 | 0.272 |
|  |  | Yes | 92 | 110 | 202 | Likelihood Ratio | 1.207 | 1 | 0.272 |
|  | Total |  | 171 | 228 | 399 | N of Valid Cases | 399 |  |  |
| Kumasi | Parent | No | 163 | 15 | 178 | Pearson Chi-Square | .209e | 1 | 0.648 |
|  |  | Yes | 107 | 8 | 115 | Likelihood Ratio | 0.212 | 1 | 0.645 |
|  | Total |  | 270 | 23 | 293 | N of Valid Cases | 293 |  |  |
| Manila | Parent | No | 112 | 25 | 137 | Pearson Chi-Square | .076f | 1 | 0.783 |
|  |  | Yes | 250 | 60 | 310 | Likelihood Ratio | 0.076 | 1 | 0.783 |
|  | Total |  | 362 | 85 | 447 | N of Valid Cases | 447 |  |  |
| Ouagadougou | Parent | No | 96 | 32 | 128 | Pearson Chi-Square | .404g | 1 | 0.525 |
|  |  | Yes | 91 | 25 | 116 | Likelihood Ratio | 0.405 | 1 | 0.524 |
|  | Total |  | 187 | 57 | 244 | N of Valid Cases | 244 |  |  |
| Port Harcourt | Parent | No | 152 | 7 | 159 | Pearson Chi-Square | .131h | 1 | 0.717 |
|  |  | Yes | 160 | 6 | 166 | Likelihood Ratio | 0.131 | 1 | 0.717 |
|  | Total |  | 312 | 13 | 325 | N of Valid Cases | 325 |  |  |
| Total | Parent | No | 850 | 231 | 1081 | Pearson Chi-Square | .036a | 1 | 0.85 |
|  |  | Yes | 938 | 250 | 1188 | Likelihood Ratio | 0.036 | 1 | 0.85 |
|  | Total |  | 1788 | 481 | 2269 | N of Valid Cases | 2269 |  |  |
| a. 0 cells (0.0%) have expected count less than 5. The minimum expected count is 229.16.  b. Computed only for a 2x2 table  c. 0 cells (0.0%) have expected count less than 5. The minimum expected count is 37.30.  d. 0 cells (0.0%) have expected count less than 5. The minimum expected count is 84.43.  e. 0 cells (0.0%) have expected count less than 5. The minimum expected count is 9.03.  f. 0 cells (0.0%) have expected count less than 5. The minimum expected count is 26.05.  g. 0 cells (0.0%) have expected count less than 5. The minimum expected count is 27.10.  h. 0 cells (0.0%) have expected count less than 5. The minimum expected count is 6.36. | | | | | | | | | |

*Table 2: Cross-tabulation of Gender during Heatwave and Non-Heatwave periods with Chi-Square Test Results Across Six Cities*

| **City** | **Cross-tabulation** | | | | | **Chi-Square Tests** | | | |
| --- | --- | --- | --- | --- | --- | --- | --- | --- | --- |
|  |  |  | Event | | Total |  |  |  | Asymptotic Significance (2-sided) |
|  |  |  | Heatwave | Non- Heatwave |  |  | Value | df |  |
| Accra | Gender | Female | 240 | 33 | 273 | Pearson Chi-Square | 6.532b | 2 | 0.038 |
|  |  | Male | 240 | 38 | 278 | Likelihood Ratio | 4.779 | 2 | 0.092 |
|  |  | Other / Prefer Not to Say | 6 | 4 | 10 | N of Valid Cases | 561 |  |  |
|  | Total |  | 486 | 75 | 561 |  |  |  |  |
| Dar es Salaam | Gender | Female | 45 | 55 | 100 | Pearson Chi-Square | 1.754c | 2 | 0.416 |
|  |  | Male | 122 | 171 | 293 | Likelihood Ratio | 1.744 | 2 | 0.418 |
|  |  | Other / Prefer Not to Say | 4 | 2 | 6 | N of Valid Cases | 399 |  |  |
|  | Total |  | 171 | 228 | 399 |  |  |  |  |
| Kumasi | Gender | Female | 145 | 13 | 158 | Pearson Chi-Square | .462d | 2 | 0.794 |
|  |  | Male | 120 | 10 | 130 | Likelihood Ratio | 0.853 | 2 | 0.653 |
|  |  | Other / Prefer Not to Say | 5 | 0 | 5 | N of Valid Cases | 293 |  |  |
|  | Total |  | 270 | 23 | 293 |  |  |  |  |
| Manila | Gender | Female | 166 | 27 | 193 | Pearson Chi-Square | 6.065e | 2 | 0.048 |
|  |  | Male | 185 | 56 | 241 | Likelihood Ratio | 6.18 | 2 | 0.046 |
|  |  | Other / Prefer Not to Say | 11 | 2 | 13 | N of Valid Cases | 447 |  |  |
|  | Total |  | 362 | 85 | 447 |  |  |  |  |
| Ouagadougou | Gender | Female | 24 | 7 | 31 | Pearson Chi-Square | .888f | 2 | 0.641 |
|  |  | Male | 153 | 45 | 198 | Likelihood Ratio | 0.822 | 2 | 0.663 |
|  |  | Other / Prefer Not to Say | 10 | 5 | 15 | N of Valid Cases | 244 |  |  |
|  | Total |  | 187 | 57 | 244 |  |  |  |  |
| Port Harcourt | Gender | Female | 139 | 7 | 146 | Pearson Chi-Square | 9.223g | 2 | 0.01 |
|  |  | Male | 166 | 4 | 170 | Likelihood Ratio | 5.541 | 2 | 0.063 |
|  |  | Other / Prefer Not to Say | 7 | 2 | 9 | N of Valid Cases | 325 |  |  |
|  | Total |  | 312 | 13 | 325 |  |  |  |  |
| Total | Gender | Female | 759 | 142 | 901 | Pearson Chi-Square | 26.502a | 2 | <.001 |
|  |  | Male | 986 | 324 | 1310 | Likelihood Ratio | 27.265 | 2 | <.001 |
|  |  | Other / Prefer Not to Say | 43 | 15 | 58 | N of Valid Cases | 2269 |  |  |
|  | Total |  | 1788 | 481 | 2269 |  |  |  |  |
| a. 0 cells (0.0%) have expected count less than 5. The minimum expected count is 12.30.  b. 1 cells (16.7%) have expected count less than 5. The minimum expected count is 1.34.  c. 2 cells (33.3%) have expected count less than 5. The minimum expected count is 2.57.  d. 2 cells (33.3%) have expected count less than 5. The minimum expected count is .39.  e. 1 cells (16.7%) have expected count less than 5. The minimum expected count is 2.47.  f. 1 cells (16.7%) have expected count less than 5. The minimum expected count is 3.50.  g. 1 cells (16.7%) have expected count less than 5. The minimum expected count is .36. | | | | | | | | | |

*Table 3: Cross-tabulation of Income during Heatwave and Non-Heatwave periods with Chi-Square Test Results Across Six Cities*

| **City** | **Cross-tabulation** | | | | | **Chi-Square Tests** | | | |
| --- | --- | --- | --- | --- | --- | --- | --- | --- | --- |
|  |  |  | Event | | Total |  |  |  |  |
|  |  |  | Heatwave | Non- Heatwave |  |  | Value | df | Asymptotic Significance (2-sided) |
| Accra | Income | <$100 | 200 | 27 | 227 | Pearson Chi-Square | 15.117b | 5 | 0.01 |
|  |  | >$4000 | 6 | 2 | 8 | Likelihood Ratio | 14.23 | 5 | 0.014 |
|  |  | $100-$499 | 102 | 21 | 123 | N of Valid Cases | 561 |  |  |
|  |  | $1500-$4000 | 16 | 7 | 23 |  |  |  |  |
|  |  | $500-$1499 | 31 | 8 | 39 |  |  |  |  |
|  |  | Don't Know | 131 | 10 | 141 |  |  |  |  |
|  | Total |  | 486 | 75 | 561 |  |  |  |  |
| Dar es Salaam | Income | <$100 | 54 | 84 | 138 | Pearson Chi-Square | 5.563c | 5 | 0.351 |
|  |  | >$4000 | 4 | 1 | 5 | Likelihood Ratio | 5.655 | 5 | 0.341 |
|  |  | $100-$499 | 20 | 35 | 55 | N of Valid Cases | 399 |  |  |
|  |  | $1500-$4000 | 2 | 2 | 4 |  |  |  |  |
|  |  | $500-$1499 | 12 | 13 | 25 |  |  |  |  |
|  |  | Don't Know | 79 | 93 | 172 |  |  |  |  |
|  | Total |  | 171 | 228 | 399 |  |  |  |  |
| Kumasi | Income | <$100 | 91 | 12 | 103 | Pearson Chi-Square | 11.033d | 5 | 0.051 |
|  |  | >$4000 | 4 | 0 | 4 | Likelihood Ratio | 13.294 | 5 | 0.021 |
|  |  | $100-$499 | 54 | 6 | 60 | N of Valid Cases | 293 |  |  |
|  |  | $1500-$4000 | 17 | 0 | 17 |  |  |  |  |
|  |  | $500-$1499 | 13 | 3 | 16 |  |  |  |  |
|  |  | Don't Know | 91 | 2 | 93 |  |  |  |  |
|  | Total |  | 270 | 23 | 293 |  |  |  |  |
| Manila | Income | <$100 | 74 | 24 | 98 | Pearson Chi-Square | 6.904e | 5 | 0.228 |
|  |  | >$4000 | 25 | 4 | 29 | Likelihood Ratio | 6.785 | 5 | 0.237 |
|  |  | $100-$499 | 70 | 14 | 84 | N of Valid Cases | 447 |  |  |
|  |  | $1500-$4000 | 21 | 9 | 30 |  |  |  |  |
|  |  | $500-$1499 | 45 | 6 | 51 |  |  |  |  |
|  |  | Don't Know | 127 | 28 | 155 |  |  |  |  |
|  | Total |  | 362 | 85 | 447 |  |  |  |  |
| Ouagadougou | Income | <$100 | 64 | 22 | 86 | Pearson Chi-Square | 9.228f | 5 | 0.1 |
|  |  | >$4000 | 3 | 1 | 4 | Likelihood Ratio | 13.587 | 5 | 0.018 |
|  |  | $100-$499 | 33 | 6 | 39 | N of Valid Cases | 244 |  |  |
|  |  | $1500-$4000 | 7 | 0 | 7 |  |  |  |  |
|  |  | $500-$1499 | 12 | 0 | 12 |  |  |  |  |
|  |  | Don't Know | 68 | 28 | 96 |  |  |  |  |
|  | Total |  | 187 | 57 | 244 |  |  |  |  |
| Port Harcourt | Income | <$100 | 97 | 0 | 97 | Pearson Chi-Square | 14.150g | 5 | 0.015 |
|  |  | >$4000 | 19 | 1 | 20 | Likelihood Ratio | 17.879 | 5 | 0.003 |
|  |  | $100-$499 | 49 | 0 | 49 | N of Valid Cases | 325 |  |  |
|  |  | $1500-$4000 | 21 | 1 | 22 |  |  |  |  |
|  |  | $500-$1499 | 29 | 1 | 30 |  |  |  |  |
|  |  | Don't Know | 97 | 10 | 107 |  |  |  |  |
|  | Total |  | 312 | 13 | 325 |  |  |  |  |
| Total | Income | <$100 | 580 | 169 | 749 | Pearson Chi-Square | 6.325a | 5 | 0.276 |
|  |  | >$4000 | 61 | 9 | 70 | Likelihood Ratio | 6.73 | 5 | 0.242 |
|  |  | $100-$499 | 328 | 82 | 410 | N of Valid Cases | 2269 |  |  |
|  |  | $1500-$4000 | 84 | 19 | 103 | a. 0 cells (0.0%) have expected count less than 5. The minimum expected count is 14.84. | | | |
|  |  | $500-$1499 | 142 | 31 | 173 | b. 2 cells (16.7%) have expected count less than 5. The minimum expected count is 1.07. | | | |
|  |  | Don't Know | 593 | 171 | 764 | c. 4 cells (33.3%) have expected count less than 5. The minimum expected count is 1.71. | | | |
|  | Total |  | 1788 | 481 | 2269 | d. 5 cells (41.7%) have expected count less than 5. The minimum expected count is .31. | | | |
|  |  |  |  |  |  | e. 0 cells (0.0%) have expected count less than 5. The minimum expected count is 5.51. | | | |
|  |  |  |  |  |  | f. 4 cells (33.3%) have expected count less than 5. The minimum expected count is .93. | | | |
|  |  |  |  |  |  | g. 6 cells (50.0%) have expected count less than 5. The minimum expected count is .80. | | | |

*Table 4: Mean Age of Participants during Heatwave and Non-Heatwave periods with Independent Samples T-tests Across Six Cities*

| **Mean Age of Participants** | | | | | | |
| --- | --- | --- | --- | --- | --- | --- |
| Events | City | | | | | |
|  | Accra | Dar es Salaam | Kumasi | Manila | Ouagadougou | Port Harcourt |
| Heatwaves | 28 | 28 | 27 | 41 | 29 | 29 |
| Non-Heatwaves | 38 | 28 | 26 | 27 | 25 | 29 |

| **Independent Samples T-test** | | | | | |
| --- | --- | --- | --- | --- | --- |
| Group Statistics |  |  |  |  |  |
|  | Events | N | Mean | Std. Deviation | Std. Error Mean |
| Age | Heatwave | 2450 | 31.48 | 13.974 | 0.282 |
|  | No Heatwave | 660 | 28.93 | 14.955 | 0.582 |

| **Independent Samples Test** | | | | | | | | | | | |
| --- | --- | --- | --- | --- | --- | --- | --- | --- | --- | --- | --- |
|  |  | Levene's Test for Equality of Variances | | t-test for Equality of Means | | |  |  |  |  |  |
|  |  | F | Sig. | t | df | Significance | | Mean Difference | Std. Error Difference | 95% Confidence Interval of the Difference | |
|  |  |  |  |  |  | 1-Sided p | 2-Sided p |  |  | Lower | Upper |
| Age | Equal variances assumed | 1.263 | 0.261 | 4.102 | 3108 | <.001 | <.001 | 2.552 | 0.622 | 1.332 | 3.772 |
|  | Equal variances not assumed |  |  | 3.945 | 990.684 | <.001 | <.001 | 2.552 | 0.647 | 1.283 | 3.822 |

| **Independent Samples Effect Sizes** | | | | | |
| --- | --- | --- | --- | --- | --- |
|  |  | Standardizer^a^ | Point Estimate | 95% Confidence Interval |  |
|  |  |  |  | Lower | Upper |
| Age | Cohen's d | 14.188 | 0.18 | 0.094 | 0.266 |
|  | Hedges' correction | 14.191 | 0.18 | 0.094 | 0.266 |
|  | Glass's delta | 14.955 | 0.171 | 0.084 | 0.257 |

^a^ The denominator used in estimating the effect sizes. Cohen's d uses the pooled standard deviation. Hedges' correction uses the pooled standard deviation, plus a correction factor. Glass's delta uses the sample standard deviation of the control (i.e., the second) group.

*Table 5: Mean Age of Children during Heatwave and Non-Heatwave periods with Independent Samples T-tests Across Six Cities*

**Mean Age of Children**

| **City** | **Heatwave (Mean Age)** | **No Heatwave (Mean Age)** |
| --- | --- | --- |
| Accra | 5.71 | 5.76 |
| Dar es Salaam | 6.3 | 5.55 |
| Kumasi | 5.58 | 8.13 |
| Manila | 8.86 | 9.5 |
| Ouagadougou | 6.58 | 7.32 |
| Port Harcourt | 7.21 | 9.17 |

**Independent T-test**

| Group Statistics |  |  |  |  |  |
| --- | --- | --- | --- | --- | --- |
|  | Event | N | Mean | Std. Deviation | Std. Error Mean |
| Child’s Age | Heatwave | 940 | 6.93 | 5.894 | 0.192 |
|  | No Heatwave | 251 | 6.87 | 5.724 | 0.361 |

| **Independent Samples Test** | | | | | | | | | | | |
| --- | --- | --- | --- | --- | --- | --- | --- | --- | --- | --- | --- |
|  |  | Levene's Test for Equality of Variances | | t-test for Equality of Means | |  |  |  |  |  |  |
|  |  | F | Sig. | t | df | Significance | | Mean Difference | Std. Error Difference | 95% Confidence Interval of the Difference | |
|  |  |  |  |  |  | 1-Sided p | 2-Sided p |  |  | Lower | Upper |
| Child’s Age | Equal variances assumed | 2.061 | 0.151 | 0.145 | 1189 | 0.442 | 0.885 | 0.06 | 0.416 | -0.756 | 0.877 |
|  | Equal variances not assumed |  |  | 0.148 | 403.023 | 0.441 | 0.883 | 0.06 | 0.409 | -0.744 | 0.865 |

| **Independent Samples Effect Sizes** | | | | | |
| --- | --- | --- | --- | --- | --- |
|  |  | Standardizer^a^ | Point Estimate | 95% Confidence Interval | |
|  |  |  |  | Lower | Upper |
| Child’s Age | Cohen's d | 5.859 | 0.01 | -0.129 | 0.15 |
|  | Hedges' correction | 5.862 | 0.01 | -0.129 | 0.149 |
|  | Glass's delta | 5.724 | 0.011 | -0.129 | 0.15 |

^a^ The denominator used in estimating the effect sizes. Cohen's d uses the pooled standard deviation. Hedges' correction uses the pooled standard deviation, plus a correction factor. Glass's delta uses the sample standard deviation of the control (i.e., the second) group.
