# Supplementary material for: Living through the heat: How urban children and young people experience and envision healthier cities
Source: PLOS Glob Public Health. 2025 Oct 29;5(10):e0004879. doi: 10.1371/journal.pgph.0004879 (PMC12571289; doi:10.1371/journal.pgph.0004879)
Supplement: S4 Table — Presents participant-reported measures of general well-being and sleep quality. (DOCX) [file pgph.0004879.s011.docx]

**Supplementary Information (S) 4: Crosstabulations and Chi-Square Test Results for Wellbeing Indicators During Heatwave and Non-Heatwave Events Across Six Study Cities**

*Table 1: Cross-tabulations and Chi-Square Tests for General Wellbeing (Feeling today and Sleep Quality)*

| **Cross-tabulation for Feeling Today** | | | | |
| --- | --- | --- | --- | --- |
| City |  |  | Event | |
|  |  |  | Heatwave | No Heatwave |
| Accra | Feeling Today | Bad | 16 | 0 |
|  |  | Good | 139 | 24 |
|  |  | Ok | 89 | 19 |
|  |  | Very bad | 9 | 1 |
|  |  | Very good | 233 | 31 |
|  | Total |  | 486 | 75 |
| Dar es Salaam | Feeling Today | Bad | 4 | 15 |
|  |  | Good | 34 | 49 |
|  |  | Ok | 14 | 27 |
|  |  | Very good | 119 | 137 |
|  | Total |  | 171 | 228 |
| Kumasi | Feeling Today | Bad | 10 | 0 |
|  |  | Good | 75 | 7 |
|  |  | Ok | 43 | 6 |
|  |  | Very bad | 16 | 0 |
|  |  | Very good | 126 | 10 |
|  | Total |  | 270 | 23 |
| Manila | Feeling Today | Bad | 17 | 3 |
|  |  | Good | 106 | 25 |
|  |  | Ok | 61 | 6 |
|  |  | Very bad | 9 | 3 |
|  |  | Very good | 169 | 48 |
|  | Total |  | 362 | 85 |
| Ouagadougou | Feeling Today | Bad | 13 | 4 |
|  |  | Good | 56 | 11 |
|  |  | Ok | 33 | 10 |
|  |  | Very bad | 2 | 0 |
|  |  | Very good | 83 | 32 |
|  | Total |  | 187 | 57 |
| Port Harcourt | Feeling Today | Bad | 10 | 0 |
|  |  | Good | 68 | 6 |
|  |  | Ok | 52 | 7 |
|  |  | Very bad | 6 | 0 |
|  |  | Very good | 176 | 0 |
|  | Total |  | 312 | 13 |
| Total | Feeling Today | Bad | 70 | 22 |
|  |  | Good | 478 | 122 |
|  |  | Ok | 292 | 75 |
|  |  | Very bad | 42 | 4 |
|  |  | Very good | 906 | 258 |
|  | Total |  | 1788 | 481 |
|  |  |  |  |  |
| **Chi-Square Tests for Feeling Today** | | | | |
| City |  | Value | df | Asymptotic Significance (2-sided) |
| Accra | Pearson Chi-Square | 5.092b | 4 | 0.278 |
|  | Likelihood Ratio | 7.111 | 4 | 0.13 |
|  | N of Valid Cases | 561 |  |  |
| Dar es Salaam | Pearson Chi-Square | 6.456c | 3 | 0.091 |
|  | Likelihood Ratio | 6.798 | 3 | 0.079 |
|  | N of Valid Cases | 399 |  |  |
| Kumasi | Pearson Chi-Square | 3.623d | 4 | 0.459 |
|  | Likelihood Ratio | 5.483 | 4 | 0.241 |
|  | N of Valid Cases | 293 |  |  |
| Manila | Pearson Chi-Square | 6.250e | 4 | 0.181 |
|  | Likelihood Ratio | 7.038 | 4 | 0.134 |
|  | N of Valid Cases | 447 |  |  |
| Ouagadougou | Pearson Chi-Square | 3.695f | 4 | 0.449 |
|  | Likelihood Ratio | 4.248 | 4 | 0.373 |
|  | N of Valid Cases | 244 |  |  |
| Port Harcourt | Pearson Chi-Square | 20.755g | 4 | <.001 |
|  | Likelihood Ratio | 24.539 | 4 | <.001 |
|  | N of Valid Cases | 325 |  |  |
| Total | Pearson Chi-Square | 5.758a | 4 | 0.218 |
|  | Likelihood Ratio | 6.68 | 4 | 0.154 |
|  | N of Valid Cases | 2269 |  |  |
| a 0 cells (0.0%) have expected count less than 5. The minimum expected count is 9.75. | | | | |
| b 2 cells (20.0%) have expected count less than 5. The minimum expected count is 1.34. | | | | |
| c 0 cells (0.0%) have expected count less than 5. The minimum expected count is 8.14. | | | | |
| d 3 cells (30.0%) have expected count less than 5. The minimum expected count is .78. | | | | |
| e 2 cells (20.0%) have expected count less than 5. The minimum expected count is 2.28. | | | | |
| f 3 cells (30.0%) have expected count less than 5. The minimum expected count is .47. | | | | |
| g 4 cells (40.0%) have expected count less than 5. The minimum expected count is .24. | | | | |
|  |  |  |  |  |
| **Cross-tabulation for Sleep Quality** | | | | |
| City |  |  | Event | |
|  |  |  | Heatwave | No Heatwave |
| Accra | Sleep Quality | Bad | 23 | 2 |
|  |  | Good | 172 | 30 |
|  |  | Ok | 105 | 18 |
|  |  | Very bad | 9 | 1 |
|  |  | Very good | 177 | 24 |
|  | Total |  | 486 | 75 |
| Dar es Salaam | Sleep Quality | Bad | 9 | 17 |
|  |  | Good | 37 | 77 |
|  |  | Ok | 12 | 28 |
|  |  | Very good | 113 | 106 |
|  | Total |  | 171 | 228 |
| Kumasi | Sleep Quality | Bad | 12 | 1 |
|  |  | Good | 85 | 8 |
|  |  | Ok | 53 | 6 |
|  |  | Very bad | 6 | 0 |
|  |  | Very good | 114 | 8 |
|  | Total |  | 270 | 23 |
| Manila | Sleep Quality | Bad | 18 | 3 |
|  |  | Good | 126 | 30 |
|  |  | Ok | 64 | 12 |
|  |  | Very bad | 7 | 2 |
|  |  | Very good | 147 | 38 |
|  | Total |  | 362 | 85 |
| Ouagadougou | Sleep Quality | Bad | 18 | 5 |
|  |  | Good | 57 | 13 |
|  |  | Ok | 38 | 10 |
|  |  | Very bad | 4 | 1 |
|  |  | Very good | 70 | 28 |
|  | Total |  | 187 | 57 |
| Port Harcourt | Sleep Quality | Bad | 12 | 1 |
|  |  | Good | 100 | 5 |
|  |  | Ok | 57 | 7 |
|  |  | Very bad | 4 | 0 |
|  |  | Very good | 139 | 0 |
|  | Total |  | 312 | 13 |
| Total | Sleep Quality | Bad | 92 | 29 |
|  |  | Good | 577 | 163 |
|  |  | Ok | 329 | 81 |
|  |  | Very bad | 30 | 4 |
|  |  | Very good | 760 | 204 |
|  | Total |  | 1788 | 481 |
|  |  |  |  |  |
|  |  |  |  |  |
| **Chi-Square Tests for Sleep Quality** | | | | |
| City |  | Value | df | Asymptotic Significance (2-sided) |
| Accra | Pearson Chi-Square | 1.628b | 4 | 0.804 |
|  | Likelihood Ratio | 1.721 | 4 | 0.787 |
|  | N of Valid Cases | 561 |  |  |
| Dar es Salaam | Pearson Chi-Square | 15.290c | 3 | 0.002 |
|  | Likelihood Ratio | 15.476 | 3 | 0.001 |
|  | N of Valid Cases | 399 |  |  |
| Kumasi | Pearson Chi-Square | 1.305d | 4 | 0.861 |
|  | Likelihood Ratio | 1.754 | 4 | 0.781 |
|  | N of Valid Cases | 293 |  |  |
| Manila | Pearson Chi-Square | 1.163e | 4 | 0.884 |
|  | Likelihood Ratio | 1.202 | 4 | 0.878 |
|  | N of Valid Cases | 447 |  |  |
| Ouagadougou | Pearson Chi-Square | 2.620f | 4 | 0.623 |
|  | Likelihood Ratio | 2.606 | 4 | 0.626 |
|  | N of Valid Cases | 244 |  |  |
| Port Harcourt | Pearson Chi-Square | 14.600g | 4 | 0.006 |
|  | Likelihood Ratio | 17.723 | 4 | 0.001 |
|  | N of Valid Cases | 325 |  |  |
| Total | Pearson Chi-Square | 3.182a | 4 | 0.528 |
|  | Likelihood Ratio | 3.434 | 4 | 0.488 |
|  | N of Valid Cases | 2269 |  |  |
| a 0 cells (0.0%) have expected count less than 5. The minimum expected count is 7.21. | | | | |
| b 2 cells (20.0%) have expected count less than 5. The minimum expected count is 1.34. | | | | |
| c 0 cells (0.0%) have expected count less than 5. The minimum expected count is 11.14. | | | | |
| d 3 cells (30.0%) have expected count less than 5. The minimum expected count is .47. | | | | |
| e 2 cells (20.0%) have expected count less than 5. The minimum expected count is 1.71. | | | | |
| f 2 cells (20.0%) have expected count less than 5. The minimum expected count is 1.17. | | | | |
| g 5 cells (50.0%) have expected count less than 5. The minimum expected count is .16. | | | | |
