# Supplementary material for: Living through the heat: How urban children and young people experience and envision healthier cities
Source: PLOS Glob Public Health. 2025 Oct 29;5(10):e0004879. doi: 10.1371/journal.pgph.0004879 (PMC12571289; doi:10.1371/journal.pgph.0004879)
Supplement: S5 Table — Lists the self-reported physical and emotional health symptoms during heatwave and non-heatwave periods. (DOCX) [file pgph.0004879.s012.docx]

**Supplementary Information (S) 5 Table: Cross-tabulation and Chi-Square Test Results for Health Symptoms During Heatwave and Non-Heatwave Periods Across Six Study Cities**

*Table 1: Cross-tabulation of Health Symptoms (Physical and Mental) and their Chi-square Tests*

| **Cross-tabulation for Itchy Eyes** | | | | | | **Chi-Square Tests** | | | |
| --- | --- | --- | --- | --- | --- | --- | --- | --- | --- |
| **City** |  |  | Event |  | Total |  |  |  | Asymptotic Significance (2-sided) |
|  |  |  | Heatwave | Non-Heatwave | |  | Value | df |  |
| Accra | Itchy Eyes | No | 323 | 60 | 383 | Pearson Chi-Square | 5.498c | 1 | 0.019 |
|  |  | Yes | 163 | 15 | 178 | Likelihood Ratio | 5.908 | 1 | 0.015 |
|  | Total |  | 486 | 75 | 561 | N of Valid Cases | 561 |  |  |
| Dar es Salaam | Itchy Eyes | No | 128 | 175 | 303 | Pearson Chi-Square | .193d | 1 | 0.66 |
|  |  | Yes | 43 | 53 | 96 | Likelihood Ratio | 0.193 | 1 | 0.661 |
|  | Total |  | 171 | 228 | 399 | N of Valid Cases | 399 |  |  |
| Kumasi | Itchy Eyes | No | 188 | 18 | 206 | Pearson Chi-Square | .756e | 1 | 0.384 |
|  |  | Yes | 82 | 5 | 87 | Likelihood Ratio | 0.799 | 1 | 0.371 |
|  | Total |  | 270 | 23 | 293 | N of Valid Cases | 293 |  |  |
| Manila | Itchy Eyes | No | 261 | 38 | 299 | Pearson Chi-Square | 23.324f | 1 | <.001 |
|  |  | Yes | 101 | 47 | 148 | Likelihood Ratio | 22.151 | 1 | <.001 |
|  | Total |  | 362 | 85 | 447 | N of Valid Cases | 447 |  |  |
| Ouagadougou | Itchy Eyes | No | 143 | 46 | 189 | Pearson Chi-Square | .448g | 1 | 0.503 |
|  |  | Yes | 44 | 11 | 55 | Likelihood Ratio | 0.46 | 1 | 0.498 |
|  | Total |  | 187 | 57 | 244 | N of Valid Cases | 244 |  |  |
| Port Harcourt | Itchy Eyes | No | 207 | 13 | 220 | Pearson Chi-Square | 6.463h | 1 | 0.011 |
|  |  | Yes | 105 | 0 | 105 | Likelihood Ratio | 10.402 | 1 | 0.001 |
|  | Total |  | 312 | 13 | 325 | N of Valid Cases | 325 |  |  |
| Total | Itchy Eyes | No | 1250 | 350 | 1600 | Pearson Chi-Square | 1.486a | 1 | 0.223 |
|  |  | Yes | 538 | 131 | 669 | Likelihood Ratio | 1.503 | 1 | 0.22 |
|  | Total |  | 1788 | 481 | 2269 | N of Valid Cases | 2269 |  |  |
|  |  |  |  |  |  |  |  |  |  |
| **Cross-tabulation for Sore Throat** | | | | | | **Chi-Square Tests** | | | |
| **City** |  |  | Event |  | Total |  |  |  |  |
|  |  |  | Heatwave | No Heatwave | |  | Value | df | Asymptotic Significance (2-sided) |
| Accra | Sore Throat | No | 379 | 63 | 442 | Pearson Chi-Square | 1.407c | 1 | 0.236 |
|  |  | Yes | 107 | 12 | 119 | Likelihood Ratio | 1.49 | 1 | 0.222 |
|  | Total |  | 486 | 75 | 561 | N of Valid Cases | 561 |  |  |
| Dar es Salaam | Sore Throat | No | 131 | 179 | 310 | Pearson Chi-Square | .204d | 1 | 0.652 |
|  |  | Yes | 40 | 49 | 89 | Likelihood Ratio | 0.203 | 1 | 0.652 |
|  | Total |  | 171 | 228 | 399 | N of Valid Cases | 399 |  |  |
| Kumasi | Sore Throat | No | 210 | 20 | 230 | Pearson Chi-Square | 1.058e | 1 | 0.304 |
|  |  | Yes | 60 | 3 | 63 | Likelihood Ratio | 1.177 | 1 | 0.278 |
|  | Total |  | 270 | 23 | 293 | N of Valid Cases | 293 |  |  |
| Manila | Sore Throat | No | 259 | 49 | 308 | Pearson Chi-Square | 6.207f | 1 | 0.013 |
|  |  | Yes | 103 | 36 | 139 | Likelihood Ratio | 5.964 | 1 | 0.015 |
|  | Total |  | 362 | 85 | 447 | N of Valid Cases | 447 |  |  |
| Ouagadougou | Sore Throat | No | 147 | 49 | 196 | Pearson Chi-Square | 1.496g | 1 | 0.221 |
|  |  | Yes | 40 | 8 | 48 | Likelihood Ratio | 1.586 | 1 | 0.208 |
|  | Total |  | 187 | 57 | 244 | N of Valid Cases | 244 |  |  |
| Port Harcourt | Sore Throat | No | 231 | 11 | 242 | Pearson Chi-Square | .734h | 1 | 0.392 |
|  |  | Yes | 81 | 2 | 83 | Likelihood Ratio | 0.814 | 1 | 0.367 |
|  | Total |  | 312 | 13 | 325 | N of Valid Cases | 325 |  |  |
| Total | Sore Throat | No | 1357 | 371 | 1728 | Pearson Chi-Square | .319a | 1 | 0.572 |
|  |  | Yes | 431 | 110 | 541 | Likelihood Ratio | 0.321 | 1 | 0.571 |
|  | Total |  | 1788 | 481 | 2269 | N of Valid Cases | 2269 |  |  |
|  |  |  |  |  |  |  |  |  |  |
| **Cross-tabulation for Cough** | | | | | | **Chi-Square Tests** | | | |
| City |  |  | Event |  | Total |  |  |  |  |
|  |  |  | Heatwave | No Heatwave | |  | Value | df | Asymptotic Significance (2-sided) |
| Accra | Cough | No | 318 | 53 | 371 | Pearson Chi-Square | .795c | 1 | 0.373 |
|  |  | Yes | 168 | 22 | 190 | Likelihood Ratio | 0.811 | 1 | 0.368 |
|  | Total |  | 486 | 75 | 561 | N of Valid Cases | 561 |  |  |
| Dar es Salaam | Cough | No | 106 | 153 | 259 | Pearson Chi-Square | 1.123d | 1 | 0.289 |
|  |  | Yes | 65 | 75 | 140 | Likelihood Ratio | 1.121 | 1 | 0.29 |
|  | Total |  | 171 | 228 | 399 | N of Valid Cases | 399 |  |  |
| Kumasi | Cough | No | 187 | 19 | 206 | Pearson Chi-Square | 1.809e | 1 | 0.179 |
|  |  | Yes | 83 | 4 | 87 | Likelihood Ratio | 1.989 | 1 | 0.158 |
|  | Total |  | 270 | 23 | 293 | N of Valid Cases | 293 |  |  |
| Manila | Cough | No | 232 | 38 | 270 | Pearson Chi-Square | 10.812f | 1 | 0.001 |
|  |  | Yes | 130 | 47 | 177 | Likelihood Ratio | 10.597 | 1 | 0.001 |
|  | Total |  | 362 | 85 | 447 | N of Valid Cases | 447 |  |  |
| Ouagadougou | Cough | No | 121 | 41 | 162 | Pearson Chi-Square | 1.022g | 1 | 0.312 |
|  |  | Yes | 66 | 16 | 82 | Likelihood Ratio | 1.044 | 1 | 0.307 |
|  | Total |  | 187 | 57 | 244 | N of Valid Cases | 244 |  |  |
| Port Harcourt | Cough | No | 171 | 12 | 183 | Pearson Chi-Square | 7.134h | 1 | 0.008 |
|  |  | Yes | 141 | 1 | 142 | Likelihood Ratio | 8.674 | 1 | 0.003 |
|  | Total |  | 312 | 13 | 325 | N of Valid Cases | 325 |  |  |
| Total | Cough | No | 1135 | 316 | 1451 | Pearson Chi-Square | .809a | 1 | 0.369 |
|  |  | Yes | 653 | 165 | 818 | Likelihood Ratio | 0.813 | 1 | 0.367 |
|  | Total |  | 1788 | 481 | 2269 | N of Valid Cases | 2269 |  |  |
|  |  |  |  |  |  |  |  |  |  |
| **Cross-tabulation for Skin Irritation** | | | | | | **Chi-Square Tests** | | | |
|  |  |  |  |  |  |  |  |  |  |
| City |  |  | Event |  | Total |  |  |  |  |
|  |  |  | Heatwave | No Heatwave | |  | Value | df | Asymptotic Significance (2-sided) |
| Accra | Skin Irritation | No | 353 | 58 | 411 | Pearson Chi-Square | .733c | 1 | 0.392 |
|  |  | Yes | 133 | 17 | 150 | Likelihood Ratio | 0.755 | 1 | 0.385 |
|  | Total |  | 486 | 75 | 561 | N of Valid Cases | 561 |  |  |
| Dar es Salaam | Skin Irritation | No | 121 | 166 | 287 | Pearson Chi-Square | .203d | 1 | 0.653 |
|  |  | Yes | 50 | 62 | 112 | Likelihood Ratio | 0.202 | 1 | 0.653 |
|  | Total |  | 171 | 228 | 399 | N of Valid Cases | 399 |  |  |
| Kumasi | Skin Irritation | No | 198 | 14 | 212 | Pearson Chi-Square | 1.646e | 1 | 0.2 |
|  |  | Yes | 72 | 9 | 81 | Likelihood Ratio | 1.545 | 1 | 0.214 |
|  | Total |  | 270 | 23 | 293 | N of Valid Cases | 293 |  |  |
| Manila | Skin Irritation | No | 266 | 42 | 308 | Pearson Chi-Square | 18.612f | 1 | <.001 |
|  |  | Yes | 96 | 43 | 139 | Likelihood Ratio | 17.563 | 1 | <.001 |
|  | Total |  | 362 | 85 | 447 | N of Valid Cases | 447 |  |  |
| Ouagadougou | Skin Irritation | No | 163 | 48 | 211 | Pearson Chi-Square | .326g | 1 | 0.568 |
|  |  | Yes | 24 | 9 | 33 | Likelihood Ratio | 0.316 | 1 | 0.574 |
|  | Total |  | 187 | 57 | 244 | N of Valid Cases | 244 |  |  |
| Port Harcourt | Skin Irritation | No | 208 | 12 | 220 | Pearson Chi-Square | 3.752h | 1 | 0.053 |
|  |  | Yes | 104 | 1 | 105 | Likelihood Ratio | 4.723 | 1 | 0.03 |
|  | Total |  | 312 | 13 | 325 | N of Valid Cases | 325 |  |  |
| Total | Skin Irritation | No | 1309 | 340 | 1649 | Pearson Chi-Square | 1.216a | 1 | 0.27 |
|  |  | Yes | 479 | 141 | 620 | Likelihood Ratio | 1.203 | 1 | 0.273 |
|  | Total |  | 1788 | 481 | 2269 | N of Valid Cases | 2269 |  |  |
|  |  |  |  |  |  |  |  |  |  |
| **Cross-tabulation for Diarrhea / Vomiting** | | | | | | **Chi-Square Tests** | | | |
| City |  |  | Event |  | Total |  |  |  |  |
|  |  |  | Heatwave | No Heatwave | |  | Value | df | Asymptotic Significance (2-sided) |
| Accra | Diarrhea / Vomiting | No | 410 | 71 | 481 | Pearson Chi-Square | 5.643c | 1 | 0.018 |
|  |  | Yes | 76 | 4 | 80 | Likelihood Ratio | 6.932 | 1 | 0.008 |
|  | Total |  | 486 | 75 | 561 | N of Valid Cases | 561 |  |  |
| Dar es Salaam | Diarrhea / Vomiting | No | 124 | 187 | 311 | Pearson Chi-Square | 5.133d | 1 | 0.023 |
|  |  | Yes | 47 | 41 | 88 | Likelihood Ratio | 5.089 | 1 | 0.024 |
|  | Total |  | 171 | 228 | 399 | N of Valid Cases | 399 |  |  |
| Kumasi | Diarrhea / Vomiting | No | 219 | 19 | 238 | Pearson Chi-Square | .031e | 1 | 0.86 |
|  |  | Yes | 51 | 4 | 55 | Likelihood Ratio | 0.032 | 1 | 0.859 |
|  | Total |  | 270 | 23 | 293 | N of Valid Cases | 293 |  |  |
| Manila | Diarrhea / Vomiting | No | 295 | 47 | 342 | Pearson Chi-Square | 26.287f | 1 | <.001 |
|  |  | Yes | 67 | 38 | 105 | Likelihood Ratio | 23.659 | 1 | <.001 |
|  | Total |  | 362 | 85 | 447 | N of Valid Cases | 447 |  |  |
| Ouagadougou | Diarrhea / Vomiting | No | 155 | 47 | 202 | Pearson Chi-Square | .006g | 1 | 0.94 |
|  |  | Yes | 32 | 10 | 42 | Likelihood Ratio | 0.006 | 1 | 0.94 |
|  | Total |  | 187 | 57 | 244 | N of Valid Cases | 244 |  |  |
| Port Harcourt | Diarrhea / Vomiting | No | 237 | 12 | 249 | Pearson Chi-Square | 1.861h | 1 | 0.172 |
|  |  | Yes | 75 | 1 | 76 | Likelihood Ratio | 2.322 | 1 | 0.128 |
|  | Total |  | 312 | 13 | 325 | N of Valid Cases | 325 |  |  |
| Total | Diarrhea / Vomiting | No | 1440 | 383 | 1823 | Pearson Chi-Square | .199a | 1 | 0.655 |
|  |  | Yes | 348 | 98 | 446 | Likelihood Ratio | 0.198 | 1 | 0.656 |
|  | Total |  | 1788 | 481 | 2269 | N of Valid Cases | 2269 |  |  |
|  |  |  |  |  |  |  |  |  |  |
| **Cross-tabulation for Heat Exhaustion** | | | | | | **Chi-Square Tests** | | | |
| City |  |  | Event |  | Total |  |  |  |  |
|  |  |  | Heatwave | No Heatwave | |  | Value | df | Asymptotic Significance (2-sided) |
| Accra | Heat Exhaustion | No | 332 | 55 | 387 | Pearson Chi-Square | .765c | 1 | 0.382 |
|  |  | Yes | 154 | 20 | 174 | Likelihood Ratio | 0.784 | 1 | 0.376 |
|  | Total |  | 486 | 75 | 561 | N of Valid Cases | 561 |  |  |
| Dar es Salaam | Heat Exhaustion | No | 104 | 133 | 237 | Pearson Chi-Square | .250d | 1 | 0.617 |
|  |  | Yes | 67 | 95 | 162 | Likelihood Ratio | 0.251 | 1 | 0.617 |
|  | Total |  | 171 | 228 | 399 | N of Valid Cases | 399 |  |  |
| Kumasi | Heat Exhaustion | No | 178 | 18 | 196 | Pearson Chi-Square | 1.456e | 1 | 0.228 |
|  |  | Yes | 92 | 5 | 97 | Likelihood Ratio | 1.557 | 1 | 0.212 |
|  | Total |  | 270 | 23 | 293 | N of Valid Cases | 293 |  |  |
| Manila | Heat Exhaustion | No | 268 | 46 | 314 | Pearson Chi-Square | 13.063f | 1 | <.001 |
|  |  | Yes | 94 | 39 | 133 | Likelihood Ratio | 12.336 | 1 | <.001 |
|  | Total |  | 362 | 85 | 447 | N of Valid Cases | 447 |  |  |
| Ouagadougou | Heat Exhaustion | No | 121 | 48 | 169 | Pearson Chi-Square | 7.806g | 1 | 0.005 |
|  |  | Yes | 66 | 9 | 75 | Likelihood Ratio | 8.548 | 1 | 0.003 |
|  | Total |  | 187 | 57 | 244 | N of Valid Cases | 244 |  |  |
| Port Harcourt | Heat Exhaustion | No | 201 | 13 | 214 | Pearson Chi-Square | 7.024h | 1 | 0.008 |
|  |  | Yes | 111 | 0 | 111 | Likelihood Ratio | 11.143 | 1 | <.001 |
|  | Total |  | 312 | 13 | 325 | N of Valid Cases | 325 |  |  |
| Total | Heat Exhaustion | No | 1204 | 313 | 1517 | Pearson Chi-Square | .878a | 1 | 0.349 |
|  |  | Yes | 584 | 168 | 752 | Likelihood Ratio | 0.872 | 1 | 0.35 |
|  | Total |  | 1788 | 481 | 2269 | N of Valid Cases | 2269 |  |  |
|  |  |  |  |  |  |  |  |  |  |
| **Cross-tabulation for Respiratory Difficulties** | | | | | | **Chi-Square Tests** | | | |
| City |  |  | Event |  | Total |  |  |  |  |
|  |  |  | Heatwave | No Heatwave | |  | Value | df | Asymptotic Significance (2-sided) |
| Accra | Respiratory Difficulties | No | 400 | 66 | 466 | Pearson Chi-Square | 1.498c | 1 | 0.221 |
|  |  | Yes | 86 | 9 | 95 | Likelihood Ratio | 1.615 | 1 | 0.204 |
|  | Total |  | 486 | 75 | 561 | N of Valid Cases | 561 |  |  |
| Dar es Salaam | Respiratory Difficulties | No | 125 | 188 | 313 | Pearson Chi-Square | 5.060d | 1 | 0.024 |
|  |  | Yes | 46 | 40 | 86 | Likelihood Ratio | 5.016 | 1 | 0.025 |
|  | Total |  | 171 | 228 | 399 | N of Valid Cases | 399 |  |  |
| Kumasi | Respiratory Difficulties | No | 222 | 21 | 243 | Pearson Chi-Square | 1.235e | 1 | 0.266 |
|  |  | Yes | 48 | 2 | 50 | Likelihood Ratio | 1.437 | 1 | 0.231 |
|  | Total |  | 270 | 23 | 293 | N of Valid Cases | 293 |  |  |
| Manila | Respiratory Difficulties | No | 288 | 44 | 332 | Pearson Chi-Square | 27.828f | 1 | <.001 |
|  |  | Yes | 74 | 41 | 115 | Likelihood Ratio | 25.331 | 1 | <.001 |
|  | Total |  | 362 | 85 | 447 | N of Valid Cases | 447 |  |  |
| Ouagadougou | Respiratory Difficulties | No | 159 | 49 | 208 | Pearson Chi-Square | .031g | 1 | 0.861 |
|  |  | Yes | 28 | 8 | 36 | Likelihood Ratio | 0.031 | 1 | 0.861 |
|  | Total |  | 187 | 57 | 244 | N of Valid Cases | 244 |  |  |
| Port Harcourt | Respiratory Difficulties | No | 246 | 13 | 259 | Pearson Chi-Square | 3.451h | 1 | 0.063 |
|  |  | Yes | 66 | 0 | 66 | Likelihood Ratio | 6.039 | 1 | 0.014 |
|  | Total |  | 312 | 13 | 325 | N of Valid Cases | 325 |  |  |
| Total | Respiratory Difficulties | No | 1440 | 381 | 1821 | Pearson Chi-Square | .421a | 1 | 0.516 |
|  |  | Yes | 348 | 100 | 448 | Likelihood Ratio | 0.417 | 1 | 0.518 |
|  | Total |  | 1788 | 481 | 2269 | N of Valid Cases | 2269 |  |  |
|  |  |  |  |  |  |  |  |  |  |
| **Cross-tabulation for Low Mood** | | | | | | **Chi-Square Tests** | | | |
| City |  |  | Event |  | Total |  |  |  |  |
|  |  |  | Heatwave | No Heatwave | |  | Value | df | Asymptotic Significance (2-sided) |
| Accra | Low Mood | No | 283 | 43 | 326 | Pearson Chi-Square | .021c | 1 | 0.883 |
|  |  | Yes | 203 | 32 | 235 | Likelihood Ratio | 0.021 | 1 | 0.884 |
|  | Total |  | 486 | 75 | 561 | N of Valid Cases | 561 |  |  |
| Dar es Salaam | Low Mood | No | 117 | 166 | 283 | Pearson Chi-Square | .912d | 1 | 0.34 |
|  |  | Yes | 54 | 62 | 116 | Likelihood Ratio | 0.908 | 1 | 0.341 |
|  | Total |  | 171 | 228 | 399 | N of Valid Cases | 399 |  |  |
| Kumasi | Low Mood | No | 165 | 16 | 181 | Pearson Chi-Square | .642e | 1 | 0.423 |
|  |  | Yes | 105 | 7 | 112 | Likelihood Ratio | 0.66 | 1 | 0.417 |
|  | Total |  | 270 | 23 | 293 | N of Valid Cases | 293 |  |  |
| Manila | Low Mood | No | 228 | 43 | 271 | Pearson Chi-Square | 4.431f | 1 | 0.035 |
|  |  | Yes | 134 | 42 | 176 | Likelihood Ratio | 4.358 | 1 | 0.037 |
|  | Total |  | 362 | 85 | 447 | N of Valid Cases | 447 |  |  |
| Ouagadougou | Low Mood | No | 133 | 44 | 177 | Pearson Chi-Square | .808g | 1 | 0.369 |
|  |  | Yes | 54 | 13 | 67 | Likelihood Ratio | 0.83 | 1 | 0.362 |
|  | Total |  | 187 | 57 | 244 | N of Valid Cases | 244 |  |  |
| Port Harcourt | Low Mood | No | 165 | 13 | 178 | Pearson Chi-Square | 11.183h | 1 | <.001 |
|  |  | Yes | 147 | 0 | 147 | Likelihood Ratio | 16.099 | 1 | <.001 |
|  | Total |  | 312 | 13 | 325 | N of Valid Cases | 325 |  |  |
| Total | Low Mood | No | 1091 | 325 | 1416 | Pearson Chi-Square | 6.931a | 1 | 0.008 |
|  |  | Yes | 697 | 156 | 853 | Likelihood Ratio | 7.037 | 1 | 0.008 |
|  | Total |  | 1788 | 481 | 2269 | N of Valid Cases | 2269 |  |  |
|  |  |  |  |  |  |  |  |  |  |
|  |  |  |  |  |  |  |  |  |  |
| **Cross-tabulation for Anxiety / Stress** | | | | | | **Chi-Square Tests** | | | |
| City |  |  | Event |  | Total |  |  |  |  |
|  |  |  | Heatwave | No Heatwave | |  | Value | df | Asymptotic Significance (2-sided) |
| Accra | Anxiety / Stress | No | 285 | 44 | 329 | Pearson Chi-Square | .000c | 1 | 0.997 |
|  |  | Yes | 201 | 31 | 232 | Likelihood Ratio | 0 | 1 | 0.997 |
|  | Total |  | 486 | 75 | 561 | N of Valid Cases | 561 |  |  |
| Dar es Salaam | Anxiety / Stress | No | 118 | 166 | 284 | Pearson Chi-Square | .688d | 1 | 0.407 |
|  |  | Yes | 53 | 62 | 115 | Likelihood Ratio | 0.686 | 1 | 0.408 |
|  | Total |  | 171 | 228 | 399 | N of Valid Cases | 399 |  |  |
| Kumasi | Anxiety / Stress | No | 151 | 14 | 165 | Pearson Chi-Square | .211e | 1 | 0.646 |
|  |  | Yes | 119 | 9 | 128 | Likelihood Ratio | 0.212 | 1 | 0.645 |
|  | Total |  | 270 | 23 | 293 | N of Valid Cases | 293 |  |  |
| Manila | Anxiety / Stress | No | 223 | 38 | 261 | Pearson Chi-Square | 8.088f | 1 | 0.004 |
|  |  | Yes | 139 | 47 | 186 | Likelihood Ratio | 7.982 | 1 | 0.005 |
|  | Total |  | 362 | 85 | 447 | N of Valid Cases | 447 |  |  |
| Ouagadougou | Anxiety / Stress | No | 132 | 43 | 175 | Pearson Chi-Square | .507g | 1 | 0.477 |
|  |  | Yes | 55 | 14 | 69 | Likelihood Ratio | 0.517 | 1 | 0.472 |
|  | Total |  | 187 | 57 | 244 | N of Valid Cases | 244 |  |  |
| Port Harcourt | Anxiety / Stress | No | 149 | 12 | 161 | Pearson Chi-Square | 9.909h | 1 | 0.002 |
|  |  | Yes | 163 | 1 | 164 | Likelihood Ratio | 11.572 | 1 | <.001 |
|  | Total |  | 312 | 13 | 325 | N of Valid Cases | 325 |  |  |
| Total | Anxiety / Stress | No | 1058 | 317 | 1375 | Pearson Chi-Square | 7.195a | 1 | 0.007 |
|  |  | Yes | 730 | 164 | 894 | Likelihood Ratio | 7.291 | 1 | 0.007 |
|  | Total |  | 1788 | 481 | 2269 | N of Valid Cases | 2269 |  |  |
|  |  |  |  |  |  |  |  |  |  |
| **Cross-tabulation for Difficulty concentrating at work / school** | | | | | | **Chi-Square Tests** | | | |
| City |  |  | Event |  | Total |  |  |  |  |
|  |  |  | Heatwave | No Heatwave | |  | Value | df | Asymptotic Significance (2-sided) |
| Accra | Difficulty concentrating at work / school | No | 327 | 54 | 381 | Pearson Chi-Square | .663c | 1 | 0.415 |
|  |  | Yes | 159 | 21 | 180 | Likelihood Ratio | 0.677 | 1 | 0.411 |
|  | Total |  | 486 | 75 | 561 | N of Valid Cases | 561 |  |  |
| Dar es Salaam | Difficulty concentrating at work / school | No | 107 | 146 | 253 | Pearson Chi-Square | .090d | 1 | 0.764 |
|  |  | Yes | 64 | 82 | 146 | Likelihood Ratio | 0.09 | 1 | 0.764 |
|  | Total |  | 171 | 228 | 399 | N of Valid Cases | 399 |  |  |
| Kumasi | Difficulty concentrating at work / school | No | 186 | 16 | 202 | Pearson Chi-Square | .005e | 1 | 0.946 |
|  |  | Yes | 84 | 7 | 91 | Likelihood Ratio | 0.005 | 1 | 0.946 |
|  | Total |  | 270 | 23 | 293 | N of Valid Cases | 293 |  |  |
| Manila | Difficulty concentrating at work / school | No | 239 | 46 | 285 | Pearson Chi-Square | 4.222f | 1 | 0.04 |
|  |  | Yes | 123 | 39 | 162 | Likelihood Ratio | 4.126 | 1 | 0.042 |
|  | Total |  | 362 | 85 | 447 | N of Valid Cases | 447 |  |  |
| Ouagadougou | Difficulty concentrating at work / school | No | 117 | 40 | 157 | Pearson Chi-Square | 1.102g | 1 | 0.294 |
|  |  | Yes | 70 | 17 | 87 | Likelihood Ratio | 1.124 | 1 | 0.289 |
|  | Total |  | 187 | 57 | 244 | N of Valid Cases | 244 |  |  |
| Port Harcourt | Difficulty concentrating at work / school | No | 194 | 13 | 207 | Pearson Chi-Square | 7.719h | 1 | 0.005 |
|  |  | Yes | 118 | 0 | 118 | Likelihood Ratio | 12.036 | 1 | <.001 |
|  | Total |  | 312 | 13 | 325 | N of Valid Cases | 325 |  |  |
| Total | Difficulty concentrating at work / school | No | 1170 | 315 | 1485 | Pearson Chi-Square | .000a | 1 | 0.983 |
|  |  | Yes | 618 | 166 | 784 | Likelihood Ratio | 0 | 1 | 0.983 |
|  | Total |  | 1788 | 481 | 2269 | N of Valid Cases | 2269 |  |  |
|  |  |  |  |  |  |  |  |  |  |
| **Cross-tabulation for Headache** | | | | | | **Chi-Square Tests** | | | |
| City |  |  | Event |  | Total |  |  |  |  |
|  |  |  | Heatwave | No Heatwave | |  | Value | df | Asymptotic Significance (2-sided) |
| Accra | Headache | No | 261 | 48 | 309 | Pearson Chi-Square | 2.784c | 1 | 0.095 |
|  |  | Yes | 225 | 27 | 252 | Likelihood Ratio | 2.827 | 1 | 0.093 |
|  | Total |  | 486 | 75 | 561 | N of Valid Cases | 561 |  |  |
| Dar es Salaam | Headache | No | 102 | 154 | 256 | Pearson Chi-Square | 2.649d | 1 | 0.104 |
|  |  | Yes | 69 | 74 | 143 | Likelihood Ratio | 2.64 | 1 | 0.104 |
|  | Total |  | 171 | 228 | 399 | N of Valid Cases | 399 |  |  |
| Kumasi | Headache | No | 152 | 16 | 168 | Pearson Chi-Square | 1.525e | 1 | 0.217 |
|  |  | Yes | 118 | 7 | 125 | Likelihood Ratio | 1.577 | 1 | 0.209 |
|  | Total |  | 270 | 23 | 293 | N of Valid Cases | 293 |  |  |
| Manila | Headache | No | 229 | 37 | 266 | Pearson Chi-Square | 11.121f | 1 | <.001 |
|  |  | Yes | 133 | 48 | 181 | Likelihood Ratio | 10.931 | 1 | <.001 |
|  | Total |  | 362 | 85 | 447 | N of Valid Cases | 447 |  |  |
| Ouagadougou | Headache | No | 110 | 32 | 142 | Pearson Chi-Square | .129g | 1 | 0.719 |
|  |  | Yes | 77 | 25 | 102 | Likelihood Ratio | 0.129 | 1 | 0.72 |
|  | Total |  | 187 | 57 | 244 | N of Valid Cases | 244 |  |  |
| Port Harcourt | Headache | No | 145 | 12 | 157 | Pearson Chi-Square | 10.499h | 1 | 0.001 |
|  |  | Yes | 167 | 1 | 168 | Likelihood Ratio | 12.151 | 1 | <.001 |
|  | Total |  | 312 | 13 | 325 | N of Valid Cases | 325 |  |  |
| Total | Headache | No | 999 | 299 | 1298 | Pearson Chi-Square | 6.125a | 1 | 0.013 |
|  |  | Yes | 789 | 182 | 971 | Likelihood Ratio | 6.178 | 1 | 0.013 |
|  | Total |  | 1788 | 481 | 2269 | N of Valid Cases | 2269 |  |  |
|  |  |  |  |  |  |  |  |  |  |
| a 0 cells (0.0%) have expected count less than 5. | | | | | | | | | |
| b Computed only for a 2x2 table | | | | | | | | | |
| c 0 cells (0.0%) have expected count less than 5. | | | | | | | | | |
| d 0 cells (0.0%) have expected count less than 5. | | | | | | | | | |
| e 0 cells (0.0%) have expected count less than 5. | | | | | | | | | |
| f 0 cells (0.0%) have expected count less than 5. | | | | | | | | | |
| g 0 cells (0.0%) have expected count less than 5. | | | | | | | | | |
| h 0 cells (0.0%) have expected count less than 5. | | | | | | | | | |
