# Supplementary material for: Living through the heat: How urban children and young people experience and envision healthier cities
Source: PLOS Glob Public Health. 2025 Oct 29;5(10):e0004879. doi: 10.1371/journal.pgph.0004879 (PMC12571289; doi:10.1371/journal.pgph.0004879)
Supplement: S7 Table — Summarizes reported levels and changes in physical activity during extreme heat periods. (DOCX) [file pgph.0004879.s014.docx]

**Supplementary Information (S) 7 Table: Cross-tabulations and Chi-Square Test Results for Physical Activity Duration During Heatwave and Non-Heatwave Events Across Six Study Cities**

*Table 1: Cross-tabulations and Chi-Square tests for Physical Activity per Event per City*

| City |  |  | Event | | Total |
| --- | --- | --- | --- | --- | --- |
|  |  |  | Heatwave | Non-Heatwave |  |
| Accra | Physical Activity | 0 Minutes | 72 | 4 | 76 |
|  |  | 1-15 Minutes | 157 | 20 | 177 |
|  |  | 16-30 Minutes | 86 | 18 | 104 |
|  |  | 31-45 Minutes | 45 | 11 | 56 |
|  |  | 46-60 Minutes | 44 | 1 | 45 |
|  |  | More than 60 Minutes | 82 | 21 | 103 |
|  | Total |  | 486 | 75 | 561 |
| Dar es Salaam | Physical Activity | 0 Minutes | 43 | 54 | 97 |
|  |  | 1-15 Minutes | 74 | 88 | 162 |
|  |  | 16-30 Minutes | 24 | 38 | 62 |
|  |  | 31-45 Minutes | 5 | 12 | 17 |
|  |  | 46-60 Minutes | 5 | 9 | 14 |
|  |  | More than 60 Minutes | 20 | 27 | 47 |
|  | Total |  | 171 | 228 | 399 |
| Kumasi | Physical Activity | 0 Minutes | 39 | 3 | 42 |
|  |  | 1-15 Minutes | 86 | 6 | 92 |
|  |  | 16-30 Minutes | 52 | 6 | 58 |
|  |  | 31-45 Minutes | 20 | 3 | 23 |
|  |  | 46-60 Minutes | 19 | 1 | 20 |
|  |  | More than 60 Minutes | 54 | 4 | 58 |
|  | Total |  | 270 | 23 | 293 |
| Manila | Physical Activity | 0 Minutes | 43 | 12 | 55 |
|  |  | 1-15 Minutes | 120 | 29 | 149 |
|  |  | 16-30 Minutes | 65 | 11 | 76 |
|  |  | 31-45 Minutes | 35 | 5 | 40 |
|  |  | 46-60 Minutes | 21 | 5 | 26 |
|  |  | More than 60 Minutes | 78 | 23 | 101 |
|  | Total |  | 362 | 85 | 447 |
| Ouagadougou | Physical Activity | 0 Minutes | 29 | 8 | 37 |
|  |  | 1-15 Minutes | 53 | 16 | 69 |
|  |  | 16-30 Minutes | 37 | 11 | 48 |
|  |  | 31-45 Minutes | 17 | 4 | 21 |
|  |  | 46-60 Minutes | 7 | 2 | 9 |
|  |  | More than 60 Minutes | 44 | 16 | 60 |
|  | Total |  | 187 | 57 | 244 |
| Port Harcourt | Physical Activity | 0 Minutes | 33 | 0 | 33 |
|  |  | 1-15 Minutes | 99 | 1 | 100 |
|  |  | 16-30 Minutes | 54 | 3 | 57 |
|  |  | 31-45 Minutes | 18 | 5 | 23 |
|  |  | 46-60 Minutes | 21 | 0 | 21 |
|  |  | More than 60 Minutes | 87 | 4 | 91 |
|  | Total |  | 312 | 13 | 325 |
| Total | Physical Activity | 0 Minutes | 259 | 81 | 340 |
|  |  | 1-15 Minutes | 589 | 160 | 749 |
|  |  | 16-30 Minutes | 318 | 87 | 405 |
|  |  | 31-45 Minutes | 140 | 40 | 180 |
|  |  | 46-60 Minutes | 117 | 18 | 135 |
|  |  | More than 60 Minutes | 365 | 95 | 460 |
|  | Total |  | 1788 | 481 | 2269 |
|  |  |  |  |  |  |
|  |  |  |  |  |  |
| **Chi-Square Tests** | |  |  |  |  |
| City |  | Value | df | Asymptotic Significance (2-sided) | |
| Accra | Pearson Chi-Square | 17.472b | 5 | 0.004 |  |
|  | Likelihood Ratio | 20.03 | 5 | 0.001 |  |
|  | N of Valid Cases | 561 |  |  |  |
| Dar es Salaam | Pearson Chi-Square | 2.596c | 5 | 0.762 |  |
|  | Likelihood Ratio | 2.655 | 5 | 0.753 |  |
|  | N of Valid Cases | 399 |  |  |  |
| Kumasi | Pearson Chi-Square | 1.908d | 5 | 0.862 |  |
|  | Likelihood Ratio | 1.781 | 5 | 0.878 |  |
|  | N of Valid Cases | 293 |  |  |  |
| Manila | Pearson Chi-Square | 3.347e | 5 | 0.647 |  |
|  | Likelihood Ratio | 3.485 | 5 | 0.626 |  |
|  | N of Valid Cases | 447 |  |  |  |
| Ouagadougou | Pearson Chi-Square | .660f | 5 | 0.985 |  |
|  | Likelihood Ratio | 0.66 | 5 | 0.985 |  |
|  | N of Valid Cases | 244 |  |  |  |
| Port Harcourt | Pearson Chi-Square | 23.716g | 5 | <.001 |  |
|  | Likelihood Ratio | 17.554 | 5 | 0.004 |  |
|  | N of Valid Cases | 325 |  |  |  |
| Total | Pearson Chi-Square | 6.628a | 5 | 0.25 |  |
|  | Likelihood Ratio | 7.159 | 5 | 0.209 |  |
|  | N of Valid Cases | 2269 |  |  |  |
| a 0 cells (0.0%) have expected count less than 5. The minimum expected count is 28.62. | | | | | |
| b 0 cells (0.0%) have expected count less than 5. The minimum expected count is 6.02. | | | | | |
| c 0 cells (0.0%) have expected count less than 5. The minimum expected count is 6.00. | | | | | |
| d 5 cells (41.7%) have expected count less than 5. The minimum expected count is 1.57. | | | | | |
| e 1 cells (8.3%) have expected count less than 5. The minimum expected count is 4.94. | | | | | |
| f 2 cells (16.7%) have expected count less than 5. The minimum expected count is 2.10. | | | | | |
| g 6 cells (50.0%) have expected count less than 5. The minimum expected count is .84. | | | | | |
