# Supplementary material for: Living through the heat: How urban children and young people experience and envision healthier cities
Source: PLOS Glob Public Health. 2025 Oct 29;5(10):e0004879. doi: 10.1371/journal.pgph.0004879 (PMC12571289; doi:10.1371/journal.pgph.0004879)
Supplement: S8 Table — Details interruptions to school, work, and caregiving activities during heatwaves. (DOCX) [file pgph.0004879.s015.docx]

**Supplementary Information (S) 8 Table: Cross-tabulations and Chi-Square Test Results for Daily Activity Disruptions During Heatwave and Non-Heatwave Events Across Six Study Cities**

*Table 1: Cross-tabulations and Chi-Square Tests for Daily Activity Disruptions*

| **Cross-tabulations for Late for School / Work** | | | | | | | **Chi-Square Tests for Late for School / Work** | | | | | | |
| --- | --- | --- | --- | --- | --- | --- | --- | --- | --- | --- | --- | --- | --- |
| City |  |  | Event | | | Total | City |  | Value | df | Asymptotic Significance (2-sided) | Exact Sig. (2-sided) | Exact Sig. (1-sided) |
|  |  |  | Heatwave | No Heatwave |  | | Accra | Pearson Chi-Square | .695c | 1 | 0.405 |  |  |
| Accra | Late for School / Work | No | 313 | 52 | | 365 |  | Continuity Correction^b^ | 0.495 | 1 | 0.482 |  |  |
|  |  | Yes | 173 | 23 | | 196 |  | Likelihood Ratio | 0.707 | 1 | 0.4 |  |  |
|  | Total |  | 486 | 75 | | 561 |  | Fisher's Exact Test | |  |  | 0.437 | 0.243 |
| Dar es Salaam | Late for School / Work | No | 113 | 154 | | 267 |  | N of Valid Cases | 561 |  |  |  |  |
|  |  | Yes | 58 | 74 | | 132 | Dar es Salaam | Pearson Chi-Square | .094d | 1 | 0.759 |  |  |
|  | Total |  | 171 | 228 | | 399 |  | Continuity Correction^b^ | 0.04 | 1 | 0.842 |  |  |
| Kumasi | Late for School / Work | No | 180 | 17 | | 197 |  | Likelihood Ratio | 0.094 | 1 | 0.759 |  |  |
|  |  | Yes | 90 | 6 | | 96 |  | Fisher's Exact Test | |  |  | 0.83 | 0.42 |
|  | Total |  | 270 | 23 | | 293 |  | N of Valid Cases | 399 |  |  |  |  |
| Manila | Late for School / Work | No | 261 | 44 | | 305 | Kumasi | Pearson Chi-Square | .505e | 1 | 0.477 |  |  |
|  |  | Yes | 101 | 41 | | 142 |  | Continuity Correction^b^ | 0.23 | 1 | 0.632 |  |  |
|  | Total |  | 362 | 85 | | 447 |  | Likelihood Ratio | 0.524 | 1 | 0.469 |  |  |
| Ouagadougou | Late for School / Work | No | 128 | 37 | | 165 |  | Fisher's Exact Test | |  |  | 0.644 | 0.323 |
|  |  | Yes | 59 | 20 | | 79 |  | N of Valid Cases | 293 |  |  |  |  |
|  | Total |  | 187 | 57 | | 244 | Manila | Pearson Chi-Square | 13.132f | 1 | <.001 |  |  |
| Port Harcourt | Late for School / Work | No | 190 | 12 | | 202 |  | Continuity Correction^b^ | 12.21 | 1 | <.001 |  |  |
|  |  | Yes | 122 | 1 | | 123 |  | Likelihood Ratio | 12.497 | 1 | <.001 |  |  |
|  | Total |  | 312 | 13 | | 325 |  | Fisher's Exact Test | |  |  | <.001 | <.001 |
| Total | Late for School / Work | No | 1185 | 316 | | 1501 |  | N of Valid Cases | 447 |  |  |  |  |
|  |  | Yes | 603 | 165 | | 768 | Ouagadougou | Pearson Chi-Square | .250g | 1 | 0.617 |  |  |
|  | Total |  | 1788 | 481 | | 2269 |  | Continuity Correction^b^ | 0.114 | 1 | 0.735 |  |  |
|  |  |  |  |  | |  |  | Likelihood Ratio | 0.247 | 1 | 0.619 |  |  |
| a 0 cells (0.0%) have expected count less than 5. The minimum expected count is 162.81. | | | | | | | | Fisher's Exact Test | |  |  | 0.63 | 0.364 |
| b Computed only for a 2x2 table | | |  |  | |  |  | N of Valid Cases | 244 |  |  |  |  |
| c 0 cells (0.0%) have expected count less than 5. The minimum expected count is 26.20. | | | | | | | Port Harcourt | Pearson Chi-Square | 5.234h | 1 | 0.022 |  |  |
| d 0 cells (0.0%) have expected count less than 5. The minimum expected count is 56.57. | | | | | | | | Continuity Correction^b^ | 3.984 | 1 | 0.046 |  |  |
| e 0 cells (0.0%) have expected count less than 5. The minimum expected count is 7.54. | | | | | | | | Likelihood Ratio | 6.514 | 1 | 0.011 |  |  |
| f 0 cells (0.0%) have expected count less than 5. The minimum expected count is 27.00. | | | | | | | | Fisher's Exact Test | |  |  | 0.021 | 0.017 |
| g 0 cells (0.0%) have expected count less than 5. The minimum expected count is 18.45. | | | | | | | | N of Valid Cases | 325 |  |  |  |  |
| h 1 cells (25.0%) have expected count less than 5. The minimum expected count is 4.92. | | | | | | | Total | Pearson Chi-Square | .057a | 1 | 0.812 |  |  |
|  |  |  |  |  | |  |  | Continuity Correction^b^ | 0.034 | 1 | 0.854 |  |  |
|  |  |  |  |  | |  |  | Likelihood Ratio | 0.057 | 1 | 0.812 |  |  |
|  |  |  |  |  | |  |  | Fisher's Exact Test | |  |  | 0.828 | 0.426 |
|  |  |  |  |  | |  |  | N of Valid Cases | 2269 |  |  |  |  |
|  |  |  |  |  | |  |  |  |  |  |  |  |  |
| **Cross-tabulations for Missed School /Work Completely** | | | | | | | **Chi-Square Tests for Missed School /Work Completely** | | | | | | |
| City |  |  | Event |  | | Total | City |  | Value | df | Asymptotic Significance (2-sided) | Exact Sig. (2-sided) | Exact Sig. (1-sided) |
|  |  |  | Heatwave | No Heatwave | | | Accra | Pearson Chi-Square | 4.247c | 1 | 0.039 |  |  |
| Accra | Missed School / Work | No | 385 | 67 | | 452 |  | Continuity Correction^b^ | 3.625 | 1 | 0.057 |  |  |
|  |  | Yes | 101 | 8 | | 109 |  | Likelihood Ratio | 4.798 | 1 | 0.028 |  |  |
|  | Total |  | 486 | 75 | | 561 |  | Fisher's Exact Test | |  |  | 0.041 | 0.024 |
| Dar es Salaam | Missed School / Work | No | 117 | 176 | | 293 |  | N of Valid Cases | 561 |  |  |  |  |
|  |  | Yes | 54 | 52 | | 106 | Dar es Salaam | Pearson Chi-Square | 3.854d | 1 | 0.05 |  |  |
|  | Total |  | 171 | 228 | | 399 |  | Continuity Correction^b^ | 3.418 | 1 | 0.065 |  |  |
| Kumasi | Missed School / Work | No | 210 | 20 | | 230 |  | Likelihood Ratio | 3.829 | 1 | 0.05 |  |  |
|  |  | Yes | 60 | 3 | | 63 |  | Fisher's Exact Test | |  |  | 0.052 | 0.033 |
|  | Total |  | 270 | 23 | | 293 |  | N of Valid Cases | 399 |  |  |  |  |
| Manila | Missed School / Work | No | 275 | 45 | | 320 | Kumasi | Pearson Chi-Square | 1.058e | 1 | 0.304 |  |  |
|  |  | Yes | 87 | 40 | | 127 |  | Continuity Correction^b^ | 0.584 | 1 | 0.445 |  |  |
|  | Total |  | 362 | 85 | | 447 |  | Likelihood Ratio | 1.177 | 1 | 0.278 |  |  |
| Ouagadougou | Missed School / Work | No | 146 | 43 | | 189 |  | Fisher's Exact Test | |  |  | 0.43 | 0.228 |
|  |  | Yes | 41 | 14 | | 55 |  | N of Valid Cases | 293 |  |  |  |  |
|  | Total |  | 187 | 57 | | 244 | Manila | Pearson Chi-Square | 17.943f | 1 | <.001 |  |  |
| Port Harcourt | Missed School / Work | No | 215 | 13 | | 228 |  | Continuity Correction^b^ | 16.829 | 1 | <.001 |  |  |
|  |  | Yes | 97 | 0 | | 97 |  | Likelihood Ratio | 16.739 | 1 | <.001 |  |  |
|  | Total |  | 312 | 13 | | 325 |  | Fisher's Exact Test | |  |  | <.001 | <.001 |
| Total | Missed School / Work | No | 1348 | 364 | | 1712 |  | N of Valid Cases | 447 |  |  |  |  |
|  |  | Yes | 440 | 117 | | 557 | Ouagadougou | Pearson Chi-Square | .174g | 1 | 0.677 |  |  |
|  | Total |  | 1788 | 481 | | 2269 |  | Continuity Correction^b^ | 0.056 | 1 | 0.813 |  |  |
|  |  |  |  |  | |  |  | Likelihood Ratio | 0.171 | 1 | 0.679 |  |  |
| a 0 cells (0.0%) have expected count less than 5. The minimum expected count is 118.08. | | | | | | | | Fisher's Exact Test | |  |  | 0.718 | 0.4 |
| b Computed only for a 2x2 table | | |  |  | |  |  | N of Valid Cases | 244 |  |  |  |  |
| c 0 cells (0.0%) have expected count less than 5. The minimum expected count is 14.57. | | | | | | | Port Harcourt | Pearson Chi-Square | 5.761h | 1 | 0.016 |  |  |
| d 0 cells (0.0%) have expected count less than 5. The minimum expected count is 45.43. | | | | | | | | Continuity Correction^b^ | 4.372 | 1 | 0.037 |  |  |
| e 1 cells (25.0%) have expected count less than 5. The minimum expected count is 4.95. | | | | | | | | Likelihood Ratio | 9.445 | 1 | 0.002 |  |  |
| f 0 cells (0.0%) have expected count less than 5. The minimum expected count is 24.15. | | | | | | | | Fisher's Exact Test | |  |  | 0.012 | 0.009 |
| g 0 cells (0.0%) have expected count less than 5. The minimum expected count is 12.85. | | | | | | | | N of Valid Cases | 325 |  |  |  |  |
| h 1 cells (25.0%) have expected count less than 5. The minimum expected count is 3.88. | | | | | | | Total | Pearson Chi-Square | .017a | 1 | 0.898 |  |  |
|  |  |  |  |  | |  |  | Continuity Correction^b^ | 0.005 | 1 | 0.945 |  |  |
|  |  |  |  |  | |  |  | Likelihood Ratio | 0.017 | 1 | 0.898 |  |  |
|  |  |  |  |  | |  |  | Fisher's Exact Test | |  |  | 0.952 | 0.475 |
|  |  |  |  |  | |  |  | N of Valid Cases | 2269 |  |  |  |  |
|  |  |  |  |  | |  |  |  |  |  |  |  |  |
| **Crosstabulation for Missed Important Meeting / Interview** | | | | | | | **Chi-Square Tests for Missed Important Meeting / Interview** | | | | | | |
| City |  |  | Event |  | | Total | City |  | Value | df | Asymptotic Significance (2-sided) | Exact Sig. (2-sided) | Exact Sig. (1-sided) |
|  |  |  | Heatwave | No Heatwave | | | Accra | Pearson Chi-Square | .417c | 1 | 0.519 |  |  |
| Accra | Missed Important Meeting / Interview | No | 400 | 64 | | 464 |  | Continuity Correction^b^ | 0.232 | 1 | 0.63 |  |  |
|  |  | Yes | 86 | 11 | | 97 |  | Likelihood Ratio | 0.432 | 1 | 0.511 |  |  |
|  | Total |  | 486 | 75 | | 561 |  | Fisher's Exact Test | |  |  | 0.623 | 0.323 |
| Dar es Salaam | Missed Important Meeting / Interview | No | 120 | 175 | | 295 |  | N of Valid Cases | 561 |  |  |  |  |
|  |  | Yes | 51 | 53 | | 104 | Dar es Salaam | Pearson Chi-Square | 2.195d | 1 | 0.138 |  |  |
|  | Total |  | 171 | 228 | | 399 |  | Continuity Correction^b^ | 1.867 | 1 | 0.172 |  |  |
| Kumasi | Missed Important Meeting / Interview | No | 220 | 20 | | 240 |  | Likelihood Ratio | 2.182 | 1 | 0.14 |  |  |
|  |  | Yes | 50 | 3 | | 53 |  | Fisher's Exact Test | |  |  | 0.167 | 0.086 |
|  | Total |  | 270 | 23 | | 293 |  | N of Valid Cases | 399 |  |  |  |  |
| Manila | Missed Important Meeting / Interview | No | 292 | 47 | | 339 | Kumasi | Pearson Chi-Square | .429e | 1 | 0.513 |  |  |
|  |  | Yes | 70 | 38 | | 108 |  | Continuity Correction^b^ | 0.139 | 1 | 0.709 |  |  |
|  | Total |  | 362 | 85 | | 447 |  | Likelihood Ratio | 0.462 | 1 | 0.497 |  |  |
| Ouagadougou | Missed Important Meeting / Interview | No | 145 | 43 | | 188 |  | Fisher's Exact Test | |  |  | 0.777 | 0.373 |
|  |  | Yes | 42 | 14 | | 56 |  | N of Valid Cases | 293 |  |  |  |  |
|  | Total |  | 187 | 57 | | 244 | Manila | Pearson Chi-Square | 24.178f | 1 | <.001 |  |  |
| Port Harcourt | Missed Important Meeting / Interview | No | 230 | 13 | | 243 |  | Continuity Correction^b^ | 22.813 | 1 | <.001 |  |  |
|  |  | Yes | 82 | 0 | | 82 |  | Likelihood Ratio | 21.902 | 1 | <.001 |  |  |
|  | Total |  | 312 | 13 | | 325 |  | Fisher's Exact Test | |  |  | <.001 | <.001 |
| Total | Missed Important Meeting / Interview | No | 1407 | 362 | | 1769 |  | N of Valid Cases | 447 |  |  |  |  |
|  |  | Yes | 381 | 119 | | 500 | Ouagadougou | Pearson Chi-Square | .109g | 1 | 0.741 |  |  |
|  | Total |  | 1788 | 481 | | 2269 |  | Continuity Correction^b^ | 0.023 | 1 | 0.88 |  |  |
|  |  |  |  |  | |  |  | Likelihood Ratio | 0.108 | 1 | 0.743 |  |  |
| a 0 cells (0.0%) have expected count less than 5. The minimum expected count is 105.99. | | | | | | | | Fisher's Exact Test | |  |  | 0.722 | 0.434 |
| b Computed only for a 2x2 table | | |  |  | |  |  | N of Valid Cases | 244 |  |  |  |  |
| c 0 cells (0.0%) have expected count less than 5. The minimum expected count is 12.97. | | | | | | | Port Harcourt | Pearson Chi-Square | 4.570h | 1 | 0.033 |  |  |
| d 0 cells (0.0%) have expected count less than 5. The minimum expected count is 44.57. | | | | | | | | Continuity Correction^b^ | 3.283 | 1 | 0.07 |  |  |
| e 1 cells (25.0%) have expected count less than 5. The minimum expected count is 4.16. | | | | | | | | Likelihood Ratio | 7.741 | 1 | 0.005 |  |  |
| f 0 cells (0.0%) have expected count less than 5. The minimum expected count is 20.54. | | | | | | | | Fisher's Exact Test | |  |  | 0.044 | 0.021 |
| g 0 cells (0.0%) have expected count less than 5. The minimum expected count is 13.08. | | | | | | | | N of Valid Cases | 325 |  |  |  |  |
| h 1 cells (25.0%) have expected count less than 5. The minimum expected count is 3.28. | | | | | | | Total | Pearson Chi-Square | 2.598a | 1 | 0.107 |  |  |
|  |  |  |  |  | |  |  | Continuity Correctionb | 2.402 | 1 | 0.121 |  |  |
|  |  |  |  |  | |  |  | Likelihood Ratio | 2.546 | 1 | 0.111 |  |  |
|  |  |  |  |  | |  |  | Fisher's Exact Test | |  |  | 0.108 | 0.062 |
|  |  |  |  |  | |  |  | N of Valid Cases | 2269 |  |  |  |  |
| **Crosstabulation for Missed Healthcare Appointment** | | | | | | | **Chi-Square Tests** | | | | | | |
| City |  |  | Event |  | | Total | City |  | Value | df | Asymptotic Significance (2-sided) | Exact Sig. (2-sided) | Exact Sig. (1-sided) |
|  |  |  | Heatwave | No Heatwave | | | Accra | Pearson Chi-Square | 5.290c | 1 | 0.021 |  |  |
| Accra | Missed Healthcare Appointment | No | 378 | 67 | | 445 |  | Continuity Correction^b^ | 4.609 | 1 | 0.032 |  |  |
|  |  | Yes | 108 | 8 | | 116 |  | Likelihood Ratio | 6.03 | 1 | 0.014 |  |  |
|  | Total |  | 486 | 75 | | 561 |  | Fisher's Exact Test | |  |  | 0.021 | 0.012 |
| Dar es Salaam | Missed Healthcare Appointment | No | 123 | 170 | | 293 |  | N of Valid Cases | 561 |  |  |  |  |
|  |  | Yes | 48 | 58 | | 106 | Dar es Salaam | Pearson Chi-Square | .347d | 1 | 0.556 |  |  |
|  | Total |  | 171 | 228 | | 399 |  | Continuity Correction^b^ | 0.225 | 1 | 0.635 |  |  |
| Kumasi | Missed Healthcare Appointment | No | 211 | 20 | | 231 |  | Likelihood Ratio | 0.346 | 1 | 0.556 |  |  |
|  |  | Yes | 59 | 3 | | 62 |  | Fisher's Exact Test | |  |  | 0.569 | 0.317 |
|  | Total |  | 270 | 23 | | 293 |  | N of Valid Cases | 399 |  |  |  |  |
| Manila | Missed Healthcare Appointment | No | 289 | 47 | | 336 | Kumasi | Pearson Chi-Square | .986e | 1 | 0.321 |  |  |
|  |  | Yes | 73 | 38 | | 111 |  | Continuity Correction^b^ | 0.528 | 1 | 0.467 |  |  |
|  | Total |  | 362 | 85 | | 447 |  | Likelihood Ratio | 1.093 | 1 | 0.296 |  |  |
| Ouagadougou | Missed Healthcare Appointment | No | 140 | 46 | | 186 |  | Fisher's Exact Test | |  |  | 0.43 | 0.241 |
|  |  | Yes | 47 | 11 | | 58 |  | N of Valid Cases | 293 |  |  |  |  |
|  | Total |  | 187 | 57 | | 244 | Manila | Pearson Chi-Square | 22.209f | 1 | <.001 |  |  |
| Port Harcourt | Missed Healthcare Appointment | No | 222 | 13 | | 235 |  | Continuity Correction^b^ | 20.914 | 1 | <.001 |  |  |
|  |  | Yes | 90 | 0 | | 90 |  | Likelihood Ratio | 20.244 | 1 | <.001 |  |  |
|  | Total |  | 312 | 13 | | 325 |  | Fisher's Exact Test | |  |  | <.001 | <.001 |
| Total | Missed Healthcare Appointment | No | 1363 | 363 | | 1726 |  | N of Valid Cases | 447 |  |  |  |  |
|  |  | Yes | 425 | 118 | | 543 | Ouagadougou | Pearson Chi-Square | .821g | 1 | 0.365 |  |  |
|  | Total |  | 1788 | 481 | | 2269 |  | Continuity Correction^b^ | 0.53 | 1 | 0.466 |  |  |
|  |  |  |  |  | |  |  | Likelihood Ratio | 0.849 | 1 | 0.357 |  |  |
| a 0 cells (0.0%) have expected count less than 5. The minimum expected count is 115.11. | | | | | | | | Fisher's Exact Test | |  |  | 0.477 | 0.236 |
| b Computed only for a 2x2 table | | |  |  | |  |  | N of Valid Cases | 244 |  |  |  |  |
| c 0 cells (0.0%) have expected count less than 5. The minimum expected count is 15.51. | | | | | | | Port Harcourt | Pearson Chi-Square | 5.186h | 1 | 0.023 |  |  |
| d 0 cells (0.0%) have expected count less than 5. The minimum expected count is 45.43. | | | | | | | | Continuity Correction^b^ | 3.846 | 1 | 0.05 |  |  |
| e 1 cells (25.0%) have expected count less than 5. The minimum expected count is 4.87. | | | | | | | | Likelihood Ratio | 8.636 | 1 | 0.003 |  |  |
| f 0 cells (0.0%) have expected count less than 5. The minimum expected count is 21.11. | | | | | | | | Fisher's Exact Test | |  |  | 0.023 | 0.013 |
| g 0 cells (0.0%) have expected count less than 5. The minimum expected count is 13.55. | | | | | | | | N of Valid Cases | 325 |  |  |  |  |
| h 1 cells (25.0%) have expected count less than 5. The minimum expected count is 3.60. | | | | | | | Total | Pearson Chi-Square | .121a | 1 | 0.728 |  |  |
|  |  |  |  |  | |  |  | Continuity Correction^b^ | 0.083 | 1 | 0.773 |  |  |
|  |  |  |  |  | |  |  | Likelihood Ratio | 0.121 | 1 | 0.728 |  |  |
|  |  |  |  |  | |  |  | Fisher's Exact Test | |  |  | 0.718 | 0.385 |
|  |  |  |  |  | |  |  | N of Valid Cases | 2269 |  |  |  |  |
|  |  |  |  |  | |  |  |  |  |  |  |  |  |
|  |  |  |  |  | |  |  |  |  |  |  |  |  |
| **Crosstabulation for Missed Meeting with Friends / Family** | | | | | | | **Chi-Square Tests for Meeting with Friends / Family** | | | | | | |
| City |  |  | Event |  | | Total | City |  | Value | df | Asymptotic Significance (2-sided) | Exact Sig. (2-sided) | Exact Sig. (1-sided) |
|  |  |  | Heatwave | No Heatwave | | | Accra | Pearson Chi-Square | 2.105c | 1 | 0.147 |  |  |
| Accra | Missed Meeting with Friends / Family | No | 350 | 60 | | 410 |  | Continuity Correction^b^ | 1.719 | 1 | 0.19 |  |  |
|  |  | Yes | 136 | 15 | | 151 |  | Likelihood Ratio | 2.22 | 1 | 0.136 |  |  |
|  | Total |  | 486 | 75 | | 561 |  | Fisher's Exact Test | |  |  | 0.163 | 0.093 |
| Dar es Salaam | Missed Meeting with Friends / Family | No | 107 | 142 | | 249 |  | N of Valid Cases | 561 |  |  |  |  |
|  |  | Yes | 64 | 86 | | 150 | Dar es Salaam | Pearson Chi-Square | .004d | 1 | 0.952 |  |  |
|  | Total |  | 171 | 228 | | 399 |  | Continuity Correction^b^ | 0 | 1 | 1 |  |  |
| Kumasi | Missed Meeting with Friends / Family | No | 189 | 19 | | 208 |  | Likelihood Ratio | 0.004 | 1 | 0.952 |  |  |
|  |  | Yes | 81 | 4 | | 85 |  | Fisher's Exact Test | |  |  | 1 | 0.518 |
|  | Total |  | 270 | 23 | | 293 |  | N of Valid Cases | 399 |  |  |  |  |
| Manila | Missed Meeting with Friends / Family | No | 266 | 41 | | 307 | Kumasi | Pearson Chi-Square | 1.636e | 1 | 0.201 |  |  |
|  |  | Yes | 96 | 44 | | 140 |  | Continuity Correction^b^ | 1.081 | 1 | 0.298 |  |  |
|  | Total |  | 362 | 85 | | 447 |  | Likelihood Ratio | 1.794 | 1 | 0.18 |  |  |
| Ouagadougou | Missed Meeting with Friends / Family | No | 122 | 36 | | 158 |  | Fisher's Exact Test | |  |  | 0.239 | 0.148 |
|  |  | Yes | 65 | 21 | | 86 |  | N of Valid Cases | 293 |  |  |  |  |
|  | Total |  | 187 | 57 | | 244 | Manila | Pearson Chi-Square | 20.395f | 1 | <.001 |  |  |
| Port Harcourt | Missed Meeting with Friends / Family | No | 195 | 13 | | 208 |  | Continuity Correction^b^ | 19.239 | 1 | <.001 |  |  |
|  |  | Yes | 117 | 0 | | 117 |  | Likelihood Ratio | 19.238 | 1 | <.001 |  |  |
|  | Total |  | 312 | 13 | | 325 |  | Fisher's Exact Test | |  |  | <.001 | <.001 |
| Total | Missed Meeting with Friends / Family | No | 1229 | 311 | | 1540 |  | N of Valid Cases | 447 |  |  |  |  |
|  |  | Yes | 559 | 170 | | 729 | Ouagadougou | Pearson Chi-Square | .083g | 1 | 0.773 |  |  |
|  | Total |  | 1788 | 481 | | 2269 |  | Continuity Correction^b^ | 0.017 | 1 | 0.897 |  |  |
|  |  |  |  |  | |  |  | Likelihood Ratio | 0.083 | 1 | 0.774 |  |  |
| a 0 cells (0.0%) have expected count less than 5. The minimum expected count is 154.54. | | | | | | | | Fisher's Exact Test | |  |  | 0.874 | 0.445 |
| b Computed only for a 2x2 table | | |  |  | |  |  | N of Valid Cases | 244 |  |  |  |  |
| c 0 cells (0.0%) have expected count less than 5. The minimum expected count is 20.19. | | | | | | | Port Harcourt | Pearson Chi-Square | 7.617h | 1 | 0.006 |  |  |
| d 0 cells (0.0%) have expected count less than 5. The minimum expected count is 64.29. | | | | | | | | Continuity Correction^b^ | 6.077 | 1 | 0.014 |  |  |
| e 0 cells (0.0%) have expected count less than 5. The minimum expected count is 6.67. | | | | | | | | Likelihood Ratio | 11.906 | 1 | <.001 |  |  |
| f 0 cells (0.0%) have expected count less than 5. The minimum expected count is 26.62. | | | | | | | | Fisher's Exact Test | |  |  | 0.005 | 0.003 |
| g 0 cells (0.0%) have expected count less than 5. The minimum expected count is 20.09. | | | | | | | | N of Valid Cases | 325 |  |  |  |  |
| h 1 cells (25.0%) have expected count less than 5. The minimum expected count is 4.68. | | | | | | | Total | Pearson Chi-Square | 2.892a | 1 | 0.089 |  |  |
|  |  |  |  |  | |  |  | Continuity Correction^b^ | 2.708 | 1 | 0.1 |  |  |
|  |  |  |  |  | |  |  | Likelihood Ratio | 2.858 | 1 | 0.091 |  |  |
|  |  |  |  |  | |  |  | Fisher's Exact Test | |  |  | 0.099 | 0.051 |
|  |  |  |  |  | |  |  | N of Valid Cases | 2269 |  |  |  |  |
|  |  |  |  |  | |  |  |  |  |  |  |  |  |
| **Crosstabulation for Not Enough Food** | | | | | | | **Chi-Square Tests** | | | | | | |
| City |  |  | Event |  | | Total | City |  | Value | df | Asymptotic Significance (2-sided) | Exact Sig. (2-sided) | Exact Sig. (1-sided) |
|  |  |  | Heatwave | No Heatwave | | | Accra | Pearson Chi-Square | 1.178c | 1 | 0.278 |  |  |
| Accra | Not Enough Food | No | 259 | 45 | | 304 |  | Continuity Correction^b^ | 0.923 | 1 | 0.337 |  |  |
|  |  | Yes | 227 | 30 | | 257 |  | Likelihood Ratio | 1.187 | 1 | 0.276 |  |  |
|  | Total |  | 486 | 75 | | 561 |  | Fisher's Exact Test | |  |  | 0.32 | 0.168 |
| Dar es Salaam | Not Enough Food | No | 112 | 150 | | 262 |  | N of Valid Cases | 561 |  |  |  |  |
|  |  | Yes | 59 | 78 | | 137 | Dar es Salaam | Pearson Chi-Square | .004d | 1 | 0.951 |  |  |
|  | Total |  | 171 | 228 | | 399 |  | Continuity Correction^b^ | 0 | 1 | 1 |  |  |
| Kumasi | Not Enough Food | No | 150 | 15 | | 165 |  | Likelihood Ratio | 0.004 | 1 | 0.951 |  |  |
|  |  | Yes | 120 | 8 | | 128 |  | Fisher's Exact Test | |  |  | 1 | 0.518 |
|  | Total |  | 270 | 23 | | 293 |  | N of Valid Cases | 399 |  |  |  |  |
| Manila | Not Enough Food | No | 246 | 42 | | 288 | Kumasi | Pearson Chi-Square | .804e | 1 | 0.37 |  |  |
|  |  | Yes | 116 | 43 | | 159 |  | Continuity Correction^b^ | 0.459 | 1 | 0.498 |  |  |
|  | Total |  | 362 | 85 | | 447 |  | Likelihood Ratio | 0.82 | 1 | 0.365 |  |  |
| Ouagadougou | Not Enough Food | No | 101 | 35 | | 136 |  | Fisher's Exact Test | |  |  | 0.392 | 0.251 |
|  |  | Yes | 86 | 22 | | 108 |  | N of Valid Cases | 293 |  |  |  |  |
|  | Total |  | 187 | 57 | | 244 | Manila | Pearson Chi-Square | 10.329f | 1 | 0.001 |  |  |
| Port Harcourt | Not Enough Food | No | 131 | 13 | | 144 |  | Continuity Correction^b^ | 9.536 | 1 | 0.002 |  |  |
|  |  | Yes | 181 | 0 | | 181 |  | Likelihood Ratio | 9.993 | 1 | 0.002 |  |  |
|  | Total |  | 312 | 13 | | 325 |  | Fisher's Exact Test | |  |  | 0.002 | 0.001 |
| Total | Not Enough Food | No | 999 | 300 | | 1299 |  | N of Valid Cases | 447 |  |  |  |  |
|  |  | Yes | 789 | 181 | | 970 | Ouagadougou | Pearson Chi-Square | .968g | 1 | 0.325 |  |  |
|  | Total |  | 1788 | 481 | | 2269 |  | Continuity Correction^b^ | 0.691 | 1 | 0.406 |  |  |
|  |  |  |  |  | |  |  | Likelihood Ratio | 0.975 | 1 | 0.323 |  |  |
|  |  |  |  |  | |  |  | Fisher's Exact Test | |  |  | 0.363 | 0.203 |
| a 0 cells (0.0%) have expected count less than 5. The minimum expected count is 205.63. | | | | | | | | N of Valid Cases | 244 |  |  |  |  |
| b Computed only for a 2x2 table | | |  |  | |  | Port Harcourt | Pearson Chi-Square | 17.021h | 1 | <.001 |  |  |
| c 0 cells (0.0%) have expected count less than 5. The minimum expected count is 34.36. | | | | | | | | Continuity Correction^b^ | 14.751 | 1 | <.001 |  |  |
| d 0 cells (0.0%) have expected count less than 5. The minimum expected count is 58.71. | | | | | | | | Likelihood Ratio | 21.848 | 1 | <.001 |  |  |
| e 0 cells (0.0%) have expected count less than 5. The minimum expected count is 10.05. | | | | | | | | Fisher's Exact Test | |  |  | <.001 | <.001 |
| f 0 cells (0.0%) have expected count less than 5. The minimum expected count is 30.23. | | | | | | | | N of Valid Cases | 325 |  |  |  |  |
| g 0 cells (0.0%) have expected count less than 5. The minimum expected count is 25.23. | | | | | | | Total | Pearson Chi-Square | 6.538a | 1 | 0.011 |  |  |
| h 0 cells (0.0%) have expected count less than 5. The minimum expected count is 5.76. | | | | | | | | Continuity Correction^b^ | 6.276 | 1 | 0.012 |  |  |
|  |  |  |  |  | |  |  | Likelihood Ratio | 6.597 | 1 | 0.01 |  |  |
|  |  |  |  |  | |  |  | Fisher's Exact Test | |  |  | 0.011 | 0.006 |
|  |  |  |  |  | |  |  | N of Valid Cases | 2269 |  |  |  |  |
|  |  |  |  |  | |  |  |  |  |  |  |  |  |
| **Cross-tabulations for No Access to Water** | | | | | | | **Chi-Square Tests for No Access to Water** | | | | | | |
| City |  |  | Event |  | | Total |  |  |  |  |  |  |  |
|  |  |  | Heatwave | No Heatwave | | | City |  | Value | df | Asymptotic Significance (2-sided) | Exact Sig. (2-sided) | Exact Sig. (1-sided) |
| Accra | No access to Water | No | 343 | 65 | | 408 | Accra | Pearson Chi-Square | 8.481c | 1 | 0.004 |  |  |
|  |  | Yes | 143 | 10 | | 153 |  | Continuity Correction^b^ | 7.689 | 1 | 0.006 |  |  |
|  | Total |  | 486 | 75 | | 561 |  | Likelihood Ratio | 9.6 | 1 | 0.002 |  |  |
| Dar es Salaam | No access to Water | No | 116 | 155 | | 271 |  | Fisher's Exact Test | |  |  | 0.003 | 0.002 |
|  |  | Yes | 55 | 73 | | 128 |  | N of Valid Cases | 561 |  |  |  |  |
|  | Total |  | 171 | 228 | | 399 | Dar es Salaam | Pearson Chi-Square | .001d | 1 | 0.975 |  |  |
| Kumasi | No access to Water | No | 195 | 16 | | 211 |  | Continuity Correction^b^ | 0 | 1 | 1 |  |  |
|  |  | Yes | 75 | 7 | | 82 |  | Likelihood Ratio | 0.001 | 1 | 0.975 |  |  |
|  | Total |  | 270 | 23 | | 293 |  | Fisher's Exact Test | |  |  | 1 | 0.53 |
| Manila | No access to Water | No | 270 | 43 | | 313 |  | N of Valid Cases | 399 |  |  |  |  |
|  |  | Yes | 92 | 42 | | 134 | Kumasi | Pearson Chi-Square | .074e | 1 | 0.785 |  |  |
|  | Total |  | 362 | 85 | | 447 |  | Continuity Correction^b^ | 0.001 | 1 | 0.976 |  |  |
| Ouagadougou | No access to Water | No | 123 | 43 | | 166 |  | Likelihood Ratio | 0.073 | 1 | 0.787 |  |  |
|  |  | Yes | 64 | 14 | | 78 |  | Fisher's Exact Test | |  |  | 0.81 | 0.476 |
|  | Total |  | 187 | 57 | | 244 |  | N of Valid Cases | 293 |  |  |  |  |
| Port Harcourt | No access to Water | No | 206 | 13 | | 219 | Manila | Pearson Chi-Square | 18.885f | 1 | <.001 |  |  |
|  |  | Yes | 106 | 0 | | 106 |  | Continuity Correction^b^ | 17.759 | 1 | <.001 |  |  |
|  | Total |  | 312 | 13 | | 325 |  | Likelihood Ratio | 17.726 | 1 | <.001 |  |  |
| Total | No access to Water | No | 1253 | 335 | | 1588 |  | Fisher's Exact Test | |  |  | <.001 | <.001 |
|  |  | Yes | 535 | 146 | | 681 |  | N of Valid Cases | 447 |  |  |  |  |
|  | Total |  | 1788 | 481 | | 2269 | Ouagadougou | Pearson Chi-Square | 1.876g | 1 | 0.171 |  |  |
|  |  |  |  |  | |  |  | Continuity Correction^b^ | 1.458 | 1 | 0.227 |  |  |
| a 0 cells (0.0%) have expected count less than 5. The minimum expected count is 144.36. | | | | | | | | Likelihood Ratio | 1.94 | 1 | 0.164 |  |  |
| b Computed only for a 2x2 table | | |  |  | |  |  | Fisher's Exact Test | |  |  | 0.196 | 0.113 |
| c 0 cells (0.0%) have expected count less than 5. The minimum expected count is 20.45. | | | | | | | | N of Valid Cases | 244 |  |  |  |  |
| d 0 cells (0.0%) have expected count less than 5. The minimum expected count is 54.86. | | | | | | | Port Harcourt | Pearson Chi-Square | 6.554h | 1 | 0.01 |  |  |
| e 0 cells (0.0%) have expected count less than 5. The minimum expected count is 6.44. | | | | | | | | Continuity Correction^b^ | 5.1 | 1 | 0.024 |  |  |
| f 0 cells (0.0%) have expected count less than 5. The minimum expected count is 25.48. | | | | | | | | Likelihood Ratio | 10.524 | 1 | 0.001 |  |  |
| g 0 cells (0.0%) have expected count less than 5. The minimum expected count is 18.22. | | | | | | | | Fisher's Exact Test | |  |  | 0.006 | 0.005 |
| h 1 cells (25.0%) have expected count less than 5. The minimum expected count is 4.24. | | | | | | | | N of Valid Cases | 325 |  |  |  |  |
|  |  |  |  |  | |  | Total | Pearson Chi-Square | .034a | 1 | 0.854 |  |  |
|  |  |  |  |  | |  |  | Continuity Correction^b^ | 0.016 | 1 | 0.899 |  |  |
|  |  |  |  |  | |  |  | Likelihood Ratio | 0.034 | 1 | 0.855 |  |  |
|  |  |  |  |  | |  |  | Fisher's Exact Test | |  |  | 0.867 | 0.448 |
|  |  |  |  |  | |  |  | N of Valid Cases | 2269 |  |  |  |  |
|  |  |  |  |  | |  |  |  |  |  |  |  |  |
| **Crosstabulation for Need for more Family Assistance** | | | | | | | **Chi-Square Tests for Need for Family Assistance** | | | | | | |
| City |  |  | Event |  | | Total | City |  | Value | df | Asymptotic Significance (2-sided) | Exact Sig. (2-sided) | Exact Sig. (1-sided) |
|  |  |  | Heatwave | No Heatwave | | | Accra | Pearson Chi-Square | 6.159c | 1 | 0.013 |  |  |
| Accra | Need for more Family Assistance | No | 256 | 51 | | 307 |  | Continuity Correction^b^ | 5.556 | 1 | 0.018 |  |  |
|  |  | Yes | 230 | 24 | | 254 |  | Likelihood Ratio | 6.319 | 1 | 0.012 |  |  |
|  | Total |  | 486 | 75 | | 561 |  | Fisher's Exact Test | |  |  | 0.017 | 0.009 |
| Dar es Salaam | Need for more Family Assistance | No | 95 | 141 | | 236 |  | N of Valid Cases | 561 |  |  |  |  |
|  |  | Yes | 76 | 87 | | 163 | Dar es Salaam | Pearson Chi-Square | 1.598d | 1 | 0.206 |  |  |
|  | Total |  | 171 | 228 | | 399 |  | Continuity Correction^b^ | 1.349 | 1 | 0.246 |  |  |
| Kumasi | Need for more Family Assistance | No | 153 | 13 | | 166 |  | Likelihood Ratio | 1.596 | 1 | 0.206 |  |  |
|  |  | Yes | 117 | 10 | | 127 |  | Fisher's Exact Test | |  |  | 0.218 | 0.123 |
|  | Total |  | 270 | 23 | | 293 |  | N of Valid Cases | 399 |  |  |  |  |
| Manila | Need for more Family Assistance | No | 241 | 39 | | 280 | Kumasi | Pearson Chi-Square | .000e | 1 | 0.989 |  |  |
|  |  | Yes | 121 | 46 | | 167 |  | Continuity Correction^b^ | 0 | 1 | 1 |  |  |
|  | Total |  | 362 | 85 | | 447 |  | Likelihood Ratio | 0 | 1 | 0.989 |  |  |
| Ouagadougou | Need for more Family Assistance | No | 88 | 31 | | 119 |  | Fisher's Exact Test | |  |  | 1 | 0.578 |
|  |  | Yes | 99 | 26 | | 125 |  | N of Valid Cases | 293 |  |  |  |  |
|  | Total |  | 187 | 57 | | 244 | Manila | Pearson Chi-Square | 12.594f | 1 | <.001 |  |  |
| Port Harcourt | Need for more Family Assistance | No | 127 | 13 | | 140 |  | Continuity Correction^b^ | 11.726 | 1 | <.001 |  |  |
|  |  | Yes | 185 | 0 | | 185 |  | Likelihood Ratio | 12.24 | 1 | <.001 |  |  |
|  | Total |  | 312 | 13 | | 325 |  | Fisher's Exact Test | |  |  | <.001 | <.001 |
| Total | Need for more Family Assistance | No | 960 | 288 | | 1248 |  | N of Valid Cases | 447 |  |  |  |  |
|  |  | Yes | 828 | 193 | | 1021 | Ouagadougou | Pearson Chi-Square | .939g | 1 | 0.333 |  |  |
|  | Total |  | 1788 | 481 | | 2269 |  | Continuity Correction^b^ | 0.668 | 1 | 0.414 |  |  |
|  |  |  |  |  | |  |  | Likelihood Ratio | 0.939 | 1 | 0.333 |  |  |
| a 0 cells (0.0%) have expected count less than 5. The minimum expected count is 216.44. | | | | | | | | Fisher's Exact Test | |  |  | 0.366 | 0.207 |
| b Computed only for a 2x2 table | | |  |  | |  |  | N of Valid Cases | 244 |  |  |  |  |
| c 0 cells (0.0%) have expected count less than 5. The minimum expected count is 33.96. | | | | | | | Port Harcourt | Pearson Chi-Square | 17.894h | 1 | <.001 |  |  |
| d 0 cells (0.0%) have expected count less than 5. The minimum expected count is 69.86. | | | | | | | | Continuity Correction^b^ | 15.558 | 1 | <.001 |  |  |
| e 0 cells (0.0%) have expected count less than 5. The minimum expected count is 9.97. | | | | | | | | Likelihood Ratio | 22.616 | 1 | <.001 |  |  |
| f 0 cells (0.0%) have expected count less than 5. The minimum expected count is 31.76. | | | | | | | | Fisher's Exact Test | |  |  | <.001 | <.001 |
| g 0 cells (0.0%) have expected count less than 5. The minimum expected count is 27.80. | | | | | | | | N of Valid Cases | 325 |  |  |  |  |
| h 0 cells (0.0%) have expected count less than 5. The minimum expected count is 5.60. | | | | | | | Total | Pearson Chi-Square | 5.857a | 1 | 0.016 |  |  |
|  |  |  |  |  | |  |  | Continuity Correction^b^ | 5.609 | 1 | 0.018 |  |  |
|  |  |  |  |  | |  |  | Likelihood Ratio | 5.893 | 1 | 0.015 |  |  |
|  |  |  |  |  | |  |  | Fisher's Exact Test | |  |  | 0.018 | 0.009 |
|  |  |  |  |  | |  |  | N of Valid Cases | 2269 |  |  |  |  |
