# Supplementary material for: Living through the heat: How urban children and young people experience and envision healthier cities
Source: PLOS Glob Public Health. 2025 Oct 29;5(10):e0004879. doi: 10.1371/journal.pgph.0004879 (PMC12571289; doi:10.1371/journal.pgph.0004879)
Supplement: S10 Table — Displays participants’ levels of concern about current and future heatwaves. (DOCX) [file pgph.0004879.s017.docx]

**Supplementary Information (S) 10 Table: Cross-tabulation and Chi-Square Tests of Participants’ Concern About Heatwaves During and Outside of Heatwave Events Across Six Study Cities**

*Table 1: Cross-tabulation and Chi-square Tests on Participants’ Concern About Heatwaves in their City*

| **Cross-tabulation** | | | | | |
| --- | --- | --- | --- | --- | --- |
| City |  |  | Event |  | Total |
|  |  |  | Heatwave | No Heatwave | |
| Accra | Concerned About Heatwaves | 0 | 79 | 9 | 88 |
|  |  | 1 | 37 | 1 | 38 |
|  |  | 2 | 17 | 0 | 17 |
|  |  | 3 | 13 | 1 | 14 |
|  |  | 4 | 7 | 1 | 8 |
|  |  | 5 | 144 | 26 | 170 |
|  |  | 6 | 15 | 1 | 16 |
|  |  | 7 | 23 | 4 | 27 |
|  |  | 8 | 14 | 1 | 15 |
|  |  | 9 | 12 | 6 | 18 |
|  |  | 10 | 125 | 25 | 150 |
|  | Total |  | 486 | 75 | 561 |
| Dar es Salaam | Concerned About Heatwaves | 0 | 70 | 91 | 161 |
|  |  | 1 | 23 | 21 | 44 |
|  |  | 2 | 4 | 1 | 5 |
|  |  | 3 | 1 | 5 | 6 |
|  |  | 4 | 1 | 1 | 2 |
|  |  | 5 | 59 | 86 | 145 |
|  |  | 6 | 1 | 1 | 2 |
|  |  | 7 | 0 | 2 | 2 |
|  |  | 8 | 1 | 2 | 3 |
|  |  | 9 | 0 | 1 | 1 |
|  |  | 10 | 11 | 17 | 28 |
|  | Total |  | 171 | 228 | 399 |
| Kumasi | Concerned About Heatwaves | 0 | 42 | 2 | 44 |
|  |  | 1 | 19 | 2 | 21 |
|  |  | 2 | 14 | 0 | 14 |
|  |  | 3 | 11 | 3 | 14 |
|  |  | 4 | 5 | 1 | 6 |
|  |  | 5 | 78 | 8 | 86 |
|  |  | 6 | 9 | 0 | 9 |
|  |  | 7 | 5 | 2 | 7 |
|  |  | 8 | 7 | 0 | 7 |
|  |  | 9 | 11 | 0 | 11 |
|  |  | 10 | 69 | 5 | 74 |
|  | Total |  | 270 | 23 | 293 |
| Manila | Concerned About Heatwaves | 0 | 65 | 21 | 86 |
|  |  | 1 | 18 | 8 | 26 |
|  |  | 2 | 8 | 1 | 9 |
|  |  | 3 | 9 | 3 | 12 |
|  |  | 4 | 6 | 3 | 9 |
|  |  | 5 | 112 | 28 | 140 |
|  |  | 6 | 5 | 1 | 6 |
|  |  | 7 | 17 | 3 | 20 |
|  |  | 8 | 27 | 2 | 29 |
|  |  | 9 | 10 | 1 | 11 |
|  |  | 10 | 85 | 14 | 99 |
|  | Total |  | 362 | 85 | 447 |
| Ouagadougou | Concerned About Heatwaves | 0 | 64 | 19 | 83 |
|  |  | 1 | 10 | 4 | 14 |
|  |  | 2 | 5 | 3 | 8 |
|  |  | 3 | 7 | 3 | 10 |
|  |  | 4 | 4 | 2 | 6 |
|  |  | 5 | 63 | 12 | 75 |
|  |  | 6 | 1 | 0 | 1 |
|  |  | 7 | 3 | 1 | 4 |
|  |  | 8 | 3 | 3 | 6 |
|  |  | 10 | 27 | 10 | 37 |
|  | Total |  | 187 | 57 | 244 |
| Port Harcourt | Concerned About Heatwaves | 0 | 50 | 1 | 51 |
|  |  | 1 | 32 | 0 | 32 |
|  |  | 2 | 15 | 7 | 22 |
|  |  | 3 | 5 | 1 | 6 |
|  |  | 4 | 4 | 0 | 4 |
|  |  | 5 | 112 | 0 | 112 |
|  |  | 6 | 5 | 0 | 5 |
|  |  | 7 | 6 | 0 | 6 |
|  |  | 8 | 3 | 2 | 5 |
|  |  | 9 | 1 | 0 | 1 |
|  |  | 10 | 79 | 2 | 81 |
|  | Total |  | 312 | 13 | 325 |
| Total | Concerned About Heatwaves | 0 | 370 | 143 | 513 |
|  |  | 1 | 139 | 36 | 175 |
|  |  | 2 | 63 | 12 | 75 |
|  |  | 3 | 46 | 16 | 62 |
|  |  | 4 | 27 | 8 | 35 |
|  |  | 5 | 568 | 160 | 728 |
|  |  | 6 | 36 | 3 | 39 |
|  |  | 7 | 54 | 12 | 66 |
|  |  | 8 | 55 | 10 | 65 |
|  |  | 9 | 34 | 8 | 42 |
|  |  | 10 | 396 | 73 | 469 |
|  | Total |  | 1788 | 481 | 2269 |

| **Chi-Square Tests** | | | | |
| --- | --- | --- | --- | --- |
| City |  | Value | df | Asymptotic Significance (2-sided) |
| Accra | Pearson Chi-Square | 17.108b | 10 | 0.072 |
|  | Likelihood Ratio | 19.75 | 10 | 0.032 |
|  | N of Valid Cases | 561 |  |  |
| Dar es Salaam | Pearson Chi-Square | 8.984c | 10 | 0.534 |
|  | Likelihood Ratio | 10.357 | 10 | 0.41 |
|  | N of Valid Cases | 399 |  |  |
| Kumasi | Pearson Chi-Square | 12.980d | 10 | 0.225 |
|  | Likelihood Ratio | 13.559 | 10 | 0.194 |
|  | N of Valid Cases | 293 |  |  |
| Manila | Pearson Chi-Square | 11.121e | 10 | 0.348 |
|  | Likelihood Ratio | 11.575 | 10 | 0.314 |
|  | N of Valid Cases | 447 |  |  |
| Ouagadougou | Pearson Chi-Square | 6.932f | 9 | 0.644 |
|  | Likelihood Ratio | 6.834 | 9 | 0.654 |
|  | N of Valid Cases | 244 |  |  |
| Port Harcourt | Pearson Chi-Square | 71.430g | 10 | <.001 |
|  | Likelihood Ratio | 40.906 | 10 | <.001 |
|  | N of Valid Cases | 325 |  |  |
| Total | Pearson Chi-Square | 31.015a | 10 | <.001 |
|  | Likelihood Ratio | 32.009 | 10 | <.001 |
|  | N of Valid Cases | 2269 |  |  |
| a 0 cells (0.0%) have expected count less than 5. The minimum expected count is 7.42. | | | | |
| b 7 cells (31.8%) have expected count less than 5. The minimum expected count is 1.07. | | | | |
| c 14 cells (63.6%) have expected count less than 5. The minimum expected count is .43. | | | | |
| d 9 cells (40.9%) have expected count less than 5. The minimum expected count is .47. | | | | |
| e 8 cells (36.4%) have expected count less than 5. The minimum expected count is 1.14. | | | | |
| f 11 cells (55.0%) have expected count less than 5. The minimum expected count is .23. | | | | |
| g 15 cells (68.2%) have expected count less than 5. The minimum expected count is .04. | | | | |

The most common concern levels during heatwaves are 5 and 10, with 32% (568 out of 1,788) and 22% (396 out of 1,788) of respondents respectively selecting these levels. This indicates that over 54% of respondents express substantial concern during actual heatwave occurrences. These findings suggest that people become significantly more conscious and possibly fearful during the actual occurrence of heatwaves, highlighting a critical need for effective risk communication and coping strategies during such events.

In contrast, during non-heatwave periods, the majority of respondents across most cities exhibit minimal concern, with 30% (143 out of 481) indicating a complete lack of concern (level 0). However, a portion of the population remains consistently worried even without heatwaves, as 15% (73 out of 481) still report the highest level of concern (level 10). This reflects a perceived sense of safety during non-heatwave periods for the majority, but it also points to a subgroup that remains apprehensive about potential heatwave risks regardless of actual events.

City-specific patterns reveal varying levels of concern both during and outside of heatwave events. In cities like Manila and Accra, moderate concern levels persist even in non-heatwave periods, with 16% and 15% of respondents, respectively, showing moderate concern. In contrast, cities like Port Harcourt and Dar es Salaam exhibit a stark contrast between event and non-event periods. In Port Harcourt, for example, only 0.6% (2 out of 325) of respondents express high concern during non-heatwave events, compared to 25% (79 out of 312) expressing high concern during heatwave events. Dar es Salaam shows a more balanced distribution, with 23% (91 out of 399) indicating no concern during non-heatwave periods, compared to 18% (70 out of 399) during heatwave events.

The chi-square tests indicate that while heatwave events significantly influence concern levels across the entire dataset, city-specific variations exist. In cities like Port Harcourt, there is a strong association between heatwave occurrences and public concern, while in others like Dar es Salaam, Kumasi, Manila, and Ouagadougou, the relationship is not statistically significant. This variability may reflect differences in local climate patterns, public awareness, or past experiences with heatwaves. Understanding these differences can help tailor risk communication strategies to better match the local context, ensuring that public health interventions are both relevant and effective.
