# Supplementary material for: Living through the heat: How urban children and young people experience and envision healthier cities
Source: PLOS Glob Public Health. 2025 Oct 29;5(10):e0004879. doi: 10.1371/journal.pgph.0004879 (PMC12571289; doi:10.1371/journal.pgph.0004879)
Supplement: S11 Table — Summarizes Children, Young People and Parents perspectives on their satisfaction on how well their cities responded to recent heatwaves. (DOCX) [file pgph.0004879.s018.docx]

**Supplementary Information (S) 11 Table: Cross-tabulation and Chi-Square Tests of Heatwave Response Satisfaction by City and Event Type**

*Table 1: Cross-tabulation and Chi-Square Tests for Heatwave Response Satisfaction per City and Event*

| **Cross-tabulation of Heatwave Response Satisfaction (HRS)** | | | | |
| --- | --- | --- | --- | --- |
| City |  |  | Event | |
|  |  |  | Heatwave | No Heatwave |
| Accra | HRS | Neutral | 167 | 29 |
|  |  | Somewhat dissatisfied | 61 | 9 |
|  |  | Somewhat satisfied | 67 | 11 |
|  |  | Very dissatisfied | 63 | 22 |
|  |  | Very Satisfied | 128 | 4 |
|  | Total |  | 486 | 75 |
| Dar es Salaam | HRS | Neutral | 37 | 49 |
|  |  | Somewhat dissatisfied | 5 | 10 |
|  |  | Very dissatisfied | 9 | 9 |
|  |  | Very Satisfied | 120 | 160 |
|  | Total |  | 171 | 228 |
| Kumasi | HRS | Neutral | 109 | 10 |
|  |  | Somewhat dissatisfied | 19 | 2 |
|  |  | Somewhat satisfied | 36 | 4 |
|  |  | Very dissatisfied | 30 | 2 |
|  |  | Very Satisfied | 76 | 5 |
|  | Total |  | 270 | 23 |
| Manila | HRS | Neutral | 116 | 18 |
|  |  | Somewhat dissatisfied | 38 | 5 |
|  |  | Somewhat satisfied | 53 | 23 |
|  |  | Very dissatisfied | 26 | 0 |
|  |  | Very Satisfied | 129 | 39 |
|  | Total |  | 362 | 85 |
| Ouagadougou | HRS | Neutral | 60 | 10 |
|  |  | Somewhat dissatisfied | 28 | 7 |
|  |  | Somewhat satisfied | 34 | 11 |
|  |  | Very dissatisfied | 6 | 3 |
|  |  | Very Satisfied | 59 | 26 |
|  | Total |  | 187 | 57 |
| Port Harcourt | HRS | Neutral | 106 | 9 |
|  |  | Somewhat dissatisfied | 31 | 1 |
|  |  | Somewhat satisfied | 35 | 1 |
|  |  | Very dissatisfied | 34 | 2 |
|  |  | Very Satisfied | 106 | 0 |
|  | Total |  | 312 | 13 |
| Total | HRS | Neutral | 595 | 125 |
|  |  | Somewhat dissatisfied | 182 | 34 |
|  |  | Somewhat satisfied | 225 | 50 |
|  |  | Very dissatisfied | 168 | 38 |
|  |  | Very Satisfied | 618 | 234 |
|  | Total |  | 1788 | 481 |
|  |  |  |  |  |
| **Chi-Square Tests** | |  |  |  |
| City |  | Value | df | Asymptotic Significance (2-sided) |
| Accra | Pearson Chi-Square | 24.071b | 4 | <.001 |
|  | Likelihood Ratio | 26.785 | 4 | <.001 |
|  | N of Valid Cases | 561 |  |  |
| Dar es Salaam | Pearson Chi-Square | .932c | 3 | 0.818 |
|  | Likelihood Ratio | 0.942 | 3 | 0.815 |
|  | N of Valid Cases | 399 |  |  |
| Kumasi | Pearson Chi-Square | .816d | 4 | 0.936 |
|  | Likelihood Ratio | 0.822 | 4 | 0.936 |
|  | N of Valid Cases | 293 |  |  |
| Manila | Pearson Chi-Square | 18.507e | 4 | <.001 |
|  | Likelihood Ratio | 22.989 | 4 | <.001 |
|  | N of Valid Cases | 447 |  |  |
| Ouagadougou | Pearson Chi-Square | 6.450f | 4 | 0.168 |
|  | Likelihood Ratio | 6.64 | 4 | 0.156 |
|  | N of Valid Cases | 244 |  |  |
| Port Harcourt | Pearson Chi-Square | 9.231g | 4 | 0.056 |
|  | Likelihood Ratio | 12.541 | 4 | 0.014 |
|  | N of Valid Cases | 325 |  |  |
| Total | Pearson Chi-Square | 32.658a | 4 | <.001 |
|  | Likelihood Ratio | 32.097 | 4 | <.001 |
|  | N of Valid Cases | 2269 |  |  |
| a 0 cells (0.0%) have expected count less than 5. The minimum expected count is 43.67. | | | | |
| b 0 cells (0.0%) have expected count less than 5. The minimum expected count is 9.36. | | | | |
| c 0 cells (0.0%) have expected count less than 5. The minimum expected count is 6.43. | | | | |
| d 3 cells (30.0%) have expected count less than 5. The minimum expected count is 1.65. | | | | |
| e 1 cells (10.0%) have expected count less than 5. The minimum expected count is 4.94. | | | | |
| f 1 cells (10.0%) have expected count less than 5. The minimum expected count is 2.10. | | | | |
| g 5 cells (50.0%) have expected count less than 5. The minimum expected count is 1.28. | | | | |

*Table* ***Error! Use the Home tab to apply 0 to the text that you want to appear here.****.2*

Accra and Manila show strong evidence that heatwave events significantly impact satisfaction levels. Port Harcourt shows a marginal effect. Dar es Salaam, Kumasi, and Ouagadougou show no significant association, suggesting that satisfaction levels remain relatively stable regardless of heatwave conditions. When considering all cities together, the results indicate a significant relationship between heatwaves and satisfaction levels. This may imply that heatwaves generally affect public satisfaction, but the effect is not uniformly observed across all cities.
