# Supplementary material for: Living through the heat: How urban children and young people experience and envision healthier cities
Source: PLOS Glob Public Health. 2025 Oct 29;5(10):e0004879. doi: 10.1371/journal.pgph.0004879 (PMC12571289; doi:10.1371/journal.pgph.0004879)
Supplement: Attachment — Submitted filename: Response to Reviewers.docx [file pgph.0004879.s021.docx]

**Response to Reviewers**

**Manuscript ID:** PGPH-D-25-01526
 **Title:** Living through the heat: How urban children and young people experience and envision healthier cities

We thank the Academic Editor and both reviewers for their careful reading of our manuscript and for their constructive feedback. We are grateful for the opportunity to revise our paper. Below, we provide a detailed point-by-point response to each comment. Reviewer comments are shown in italics, followed by our responses and a note of changes made in the revised manuscript. Page and paragraph numbers refer to the tracked-changes version.

**Reviewer #1 (Prof. Greg Mews)**

**Comment 1:** The study failed to address ethics approval. A sentence would be useful to highlight which institution granted permission.

**Response:** We agree with the reviewer’s observation and have added a clear statement on ethics approval. In the Methods section (p. 13, Lines 223-225), we now state: “Ethical approval for this study was granted by the London School of Hygiene & Tropical Medicine Research Ethics Committee (Ref: 31044), ensuring that participants’ rights, dignity, and confidentiality were protected.”

**Comment 2:** In terms of the selection of cities, it is worth highlighting that urban form, density, and size were not considered. As the heat island effects of cities due to the number of hard surfaces are usually higher than those of the surrounding area, it needs to be made clear why this was not considered.

**Response:** Thank you for this comment. We have clarified in the Methods (Study Design and Setting) section that cities were selected because they experienced verified heatwave events, not based on their urban form, density, or size (p. 8, Lines 125–129). In addition, we added a subsection (Study area) that describes each city’s urban form, density, and size, and explains how these features relate to urban heat island effects. A locator map has also been included to show the position of the six study cities (p. 9, Lines 140–181).

**Comment 3:** regarding engagement methods, it would be beneficial to elaborate further on why a survey was chosen as the preferred method over others tailored to the user group. It is briefly mentioned in the limitations, but it deserves more attention, as there is substantial evidence in the area of participation with and for children and young people that other methods allow for much richer and nuanced insights into environmental perception.

**Response:** Thank you for this helpful comment. We have expanded the Introduction to explain why a digital survey was chosen over participatory approaches. Specifically, we note that while participatory methods provide rich insights, they are time-intensive and not feasible for rapid, cross-city research during acute events. The survey allowed real-time data collection within 24 hours of event verification (p. 6, Lines 81-94). And also in the methods (study design and setting) (p.8; Lines 122-124).

**Comment 4:** Furthermore, the study has significant limitations due to the complexity of urban systems and the existence of more appropriate methods to investigate the nexus through systems approaches.

**Response**: we have revised the Limitations section to explicitly acknowledge this limitation and to highlight that systems-based or mixed-methods approaches may be more appropriate for investigating these interconnections in depth (p. 16, Lines 289-292). This suggestion was also added in the recommendation for future research (p.39, Lines 670-677).

**Comment 5:** Care must be taken around the conclusion on sustainable and healthy cities; some cross connections deserve more clarity, especially given that food system design (I assume this includes drinking as well) is one important factor in the data findings. Yet this was not addressed in the suggestions.

**Response:** We appreciate this helpful comment. We revised the Discussion (Implications for Policy and Practice) to use more cautious language (“could prioritize” instead of “should prioritize”). We also added reference to food systems and safe drinking water, and noted that participatory and systems-oriented approaches are important for addressing cross-sectoral connections (p. 37 & p.38, Lines 639–648 & 657-661). Additionally, in the Conclusion, we now explicitly refer to food and water systems, alongside green/blue infrastructure, health care, and family support, as essential components of sustainable and healthy cities (p. 39, Lines 679–690).

**Comment 6:** The paper mentions green infrastructure, yet despite mentioning water, you might also wish to include blue infrastructure. However, there are studies on bioclimate that found that water bodies, under particular circumstances, can amplify heat effects and impact human health in other ways. Tipping ecology, including more mosquito- and vector-borne diseases, etc. Surface colours and material composition determine human comfort in different parts of those cities too.

**Response:** We thank the reviewer for this suggestion. In the “Implications for Policy and Practice,” we now include blue infrastructure alongside green infrastructure and note both its cooling potential and related risks, such as mosquito- and water-borne disease. We also added that surface colour and material composition affect human comfort and should be considered in urban design (p. 37, lines 637–648).

**Comment 7:** While the study acknowledges limitations in terms of SES, given the sample size, it might be worth focusing on the responses of participants from such communities. Perhaps this might be worth future research in combination with different methods. Hence, I suggest reevaluating the study's strengths and limitations more critically from an urban and participatory research perspective on vulnerable population groups, as the limitations outweigh the benefits of the method in relation to the theme of inquiry.

**Response:** We thank the reviewer for this valuable comment. The “Limitations” section has been expanded to note that digital surveys likely underrepresented CYP from lower socioeconomic groups and cannot provide the depth of participatory or community-based methods (p. 16, Lines 277–292). In the “Recommendations for Future Research” section, we now recommend combining these methods and conducting city-level analyses to better capture the experiences of vulnerable groups (p. 39, Lines 657–661).

**Comment 8:** Equally, I suggest caution around the recommendation for policy, but highlight that other methods might achieve more meaningful and nuanced outcomes and impact. Socio-cultural aspects of place indicate that each of those cities is quite heterogeneous, and bio-climatic conditions can vary from place to place.

**Response:** We appreciate this comment. In the “Implications for Policy and Practice,” we now emphasize that policies must be adapted to the socio-cultural and climatic contexts of individual cities. We also note that participatory and systems-oriented approaches can provide more nuanced insights, ensuring policies are context-specific and meaningful (p. 39, Lines 657–661).

**Reviewer #2 (Anonymous)**

**Comment 1:** the statement in the introduction "we know little about how [children] experience their changing urban environments, especially during extreme events" is not strictly true. Please see these references:

Salima Meherali, Yared Asmare Aynalem, Saba Un Nisa, Megan Kennedy, Bukola Salami, Samuel Adjorlolo, Parveen Ali, Kênia Lara Silva, Lydia Aziato, Solina Richter, Zohra S Lassi - Impact of climate change on child outcomes: an evidence gap map review: BMJ Paediatrics Open 2024;8:e002592.

https://futureofchildren.princeton.edu/sites/g/files/toruqf2411/files/media/children_and_climate_change_26_1_full_journal.pdf

https://www.austlii.edu.au/au/journals/AUJlEmMgmt/2020/26.pdf

**Response:** Thank you for pointing this out. We have revised the introduction to acknowledge existing evidence on children and climate change, citing the suggested references (p.4, Lines 44-53).

**Comment 2:** Provide a citation for: “A child born today risks living in a world up to four degrees Celsius warmer…”

**Response:** We have now added the Lancet Countdown reference (Watts et al., 2019) in the Introduction, p.4, Lines 51–53.

**Comment 3:** The paper misses out on discussing the literarure on the link between heat waves and heat stress. And more specifically, on heat stress both during and after heatwaves, which is driven by a complex interplay of climatic, infrastructural, and socio-environmental factors. These elements not only intensify the immediate thermal burden but also prolong discomfort and health risks well beyond the peak of a heatwave. Because this inadequate problem identification, I feel the paper lacks depth and understanding of what heat stress is and how that impacts health and well-being. For example, there is no discussion on how cities suffer more due to High Ambient and Surface Temperatures, Urban Heat Island (UHI) Effect, Relative Humidity, Reduced Wind Flow and Sky Visibility, Limited Green and Blue Infrastructure etc. Most importantly,
Heat stress is not restricted to heat waves. This is perhaps critical in explaining some of the study findings that found no difference in sleep patterns or physical activity levels during or after the heat waves. Right now the study lacks explanatory power due to inadequate context setting.

**Response:** Thank you for this suggestion. We have expanded both the introduction (p.4-5; Lines 67-73) and the discussion (p.33, Lines 545-606) to more clearly frame heat stress beyond the context of heatwaves, incorporating consideration such as environmental , infrastructural, and socio-economical factors.

**Comment 4:** The limitations of the study should be discussed in the methods section and not at the end. I really missed any discussion of whether the periods chosen as heat waves are perceived as such in the study cities. Were these recognized as "heatwaves" by the local governments? Because that has a psychological impact in people's perception of heatwaves.

**Response**: We have moved the discussion of study limitations to the Methods (Study Limitations, p. 16; Lines 277–292) as requested. We also clarified in Methods (Study Design & Setting, p. 7; Lines 117–120) that the heatwave periods in Manila and Accra were formally recognized by national meteorological agencies, while in the other cities events were defined using our percentile-based definition. We also added a paragraph in the Discussion (p.36; Lines 619-622) to address the issue of local recognition of heatwaves.

**Comment 5:** A major weakess of the design is that the study did not follow the same participants over time, making it difficult to assess whether changes in outcomes were directly linked to heatwave exposure. The study may have underestimated the true impact of heatwaves on the most vulnerable populations due to the exclusion of those without internet access or those less likely to participate in online surveys. Moreover, sample sizes varied across cities, with smaller and uneven samples during non-heatwave periods, potentially influencing the findings and limiting the generalizability of results.

**Response:** These limitations are acknowledged in the Methods (Study Limitations, p. 16; Lines 277–292)

**Comment 6:** Add background information on selected cities (urbanization, development, climate burdens).
 **Response:** We have added background information on the study cities in the introduction (P.6; Lines 95–105) and added a sub-section in the study area (p.9; Lines 141-181).

**Comment 7:** The paper needs repositioning with greater engagement with literature and deeper discussion of results.
**Response:** Thank you for this comment. We have strengthened the paper by engaging more with the literature and expanding the introduction (p.4; Lines 44-109) and discussion (p.32; Lines 545-626) sections.

**Closing**
We hope these revisions address the reviewers’ concerns and have improved the clarity, depth, and rigor of the manuscript. We thank the reviewers and editor once again for their valuable input.

Sincerely,
 Constance Bwire (on behalf of all co-authors)
